# Chloroplast genome analyses of *Caragana arborescens* and *Caragana opulens*

**DOI:** 10.1186/s12863-024-01202-4

**Published:** 2024-02-09

**Authors:** LiE Liu, Hongyan Li, Jiaxin Li, Xinjuan Li, Na Hu, Honglun Wang, Wu Zhou

**Affiliations:** 1 School of Ecological and Environmental Engineering, Qinghai University, Xining, 810016 China; 2 Key Laboratory of Tibetan Medicine Research, Northwest Institute of Plateau Biology, Chinese Academy of Sciences, Xining, 810008 China

**Keywords:** *Caragana arborescens*, *Caragana opulens*, Plastid genomes, Comparative genomics, Phylogenetic analysis

## Abstract

**Background:**

Numerous species within the genus *Caragana* have high ecological and medicinal value. However, species identification based on morphological characteristics is quite complicated in the genus. To address this issue, we analyzed complete plastid genome data for the genus.

**Results:**

We obtained chloroplast genomes of two species, *Caragana arborescens* and *Caragana opulens*, using Illumina sequencing technology, with lengths of 129,473 bp and 132,815 bp, respectively. The absence of inverted repeat sequences in the two species indicated that they could be assigned to the inverted repeat-lacking clade (IRLC). The genomes included 111 distinct genes (4 rRNA genes, 31 tRNA genes, and 76 protein-coding genes). In addition, 16 genes containing introns were identified in the two genomes, the majority of which contained a single intron. Repeat analyses revealed 129 and 229 repeats in *C. arborescens* and *C. opulens*, respectively. *C. arborescens* and *C. opulens* genomes contained 277 and 265 simple sequence repeats, respectively. The two *Caragana* species exhibited similar codon usage patterns. *rpl20-clpP, rps19-rpl2*, and *rpl23-ycf2* showed the highest nucleotide diversity (pi). In an analysis of sequence divergence, certain intergenic regions (*matK*-*rbcL*, *psbM*-*petN*, *atpA*-*psbI*, *petA*-*psbL*, *psbE*-*petL*, and *rps7*-*rps12*) were highly variable. A phylogenetic analysis showed that *C. arborescens* and *C. opulens* were related and clustered together with four other *Caragana* species. The genera *Astragalus* and *Caragana* were relatively closely related.

**Conclusions:**

The present study provides valuable information about the chloroplast genomes of *C. arborescens* and *C. opulens* and lays a foundation for future phylogenetic research and molecular marker development.

**Supplementary Information:**

The online version contains supplementary material available at 10.1186/s12863-024-01202-4.

## Background

The genus *Caragana*, which belongs to the subfamily Papilionoideae of the family Fabaceae, includes approximately 100 species, primarily found in arid and semiarid regions of Asia and Europe [1]. The majority of plants in this genus can withstand adverse environmental conditions, including sterile soil, drought, cold, high temperatures, strong winds, and insect and disease damage [1]. China is home to 66 species, 32 of which are endemic. In China, these species are primarily found at high altitudes and in harsh environments, such as shady and semi-shady areas in the northwest, southwest, northeast, and north [2]. *Caragana* is a deciduous undershrub with extensive adaptability and strong stress resistance, including resistance to wind; it also contributes to sand fixation [3]. In addition, the majority of species in the genus can fix nitrogen via nodules, thereby enhancing soil fertility and preventing dust cyclones and land desertification [4]. *Caragana arborescens*, also known as Siberian pea shrub, is typically found in Northeast China, North China, and Northwest China [5, 6]. It has a height of 4–5 m. The species, which blooms in May with yellow flowers and pods that mature in midsummer, is typically used as foliage and for garden decoration [7]. *Caragana opulens* is a shrub with a yellow corolla that thrives in the hills up to 3400 m above sea level in North China, Northwest China, and Southwest China and is distributed throughout these regions [8]. In addition, previous research has demonstrated that numerous species of this genus possess outstanding pharmacological properties, including protective effects against cancer, HIV, rheumatoid arthritis, and hypertension [1, 9, 10]. *C. arborescens* has been documented in traditional Chinese medicine and is a significant Mongolian medicine used to treat pulmonary hemorrhage and rheumatism [1].

Chloroplast genome data for *Caragana* plants are extremely limited, with only 14 reports to date. Although the phylogenetic relationships of *Caragana* plants have been studied based on nuclear ITS and plastid *matK*, *trnL-F* and *psbA-trnH* marker sequence data, the resolution of evolutionary analyses is relatively low, and there are unresolved issues regarding the classification of some medicinal plants, such as *Caragana changduensis*, *Caragana frutex* and *Caragana polourensis* [2, 11–13]. Therefore, it is crucial to discover a precise and convenient markers for identifying *Caragana* plants.

Our understanding of chloroplasts has improved substantially over the past decade, with research on their origin, structure, evolution, and genetic engineering [14–16]. It is hypothesized that the cp. is derived from bacterial endosymbiosis in eukaryotes; it is the site of photosynthesis in plants [17]. The chloroplast possesses its own DNA (cpDNA) and genetic system and exist as covalent double-stranded circular DNA in most species [18, 19]. With the rapid advancement of sequencing technology, scientists have discovered that the chloroplast genome contains effective molecular markers, facilitating the precise identification of species. The chloroplast genome is optimal for molecular identification, phylogenetic analyses, and species conservation research [20]. Unlike the nuclear genome, the chloroplast genome is characterized by unisexual inheritance, a simple structure, and more gene copies [19, 21]. Typically, the chloroplast genome is maternally inherited in angiosperms [22]. Its structure is comparatively stable and consists of a large single copy (LSC) and a small single copy (SSC) region separated by two inverted repeats (IRs) [23]. An inverted repeat-lacking clade (IRLC) has been described in leguminous plants [24–28]. Eight species of *Caragana* plants in the IRLC have been identified [2, 12, 13, 29]. With the refinement and expansion of *Caragana* chloroplast genome data, the genus will presumably represent a broad IRLC spectral system for scientific investigation. Moreover, chloroplast genome sequence data offer reliable information for analyses of genetic and phylogenetic relationships and population genomics [29–31].

In this study, the complete chloroplast genomes of *C. arborescens* and *C. opulens* were obtained using Illumina sequencing technology, and their structural properties and phylogenetic relationships were elucidated. This study enriches the chloroplast genomic database of *Caragana*, which is anticipated to serve as a foundation for systematic research in evolutionary biology and the protection and utilization of *Caragana* germplasm.

## Results

### Chloroplast genome assembly and features

**Fig. 1:**
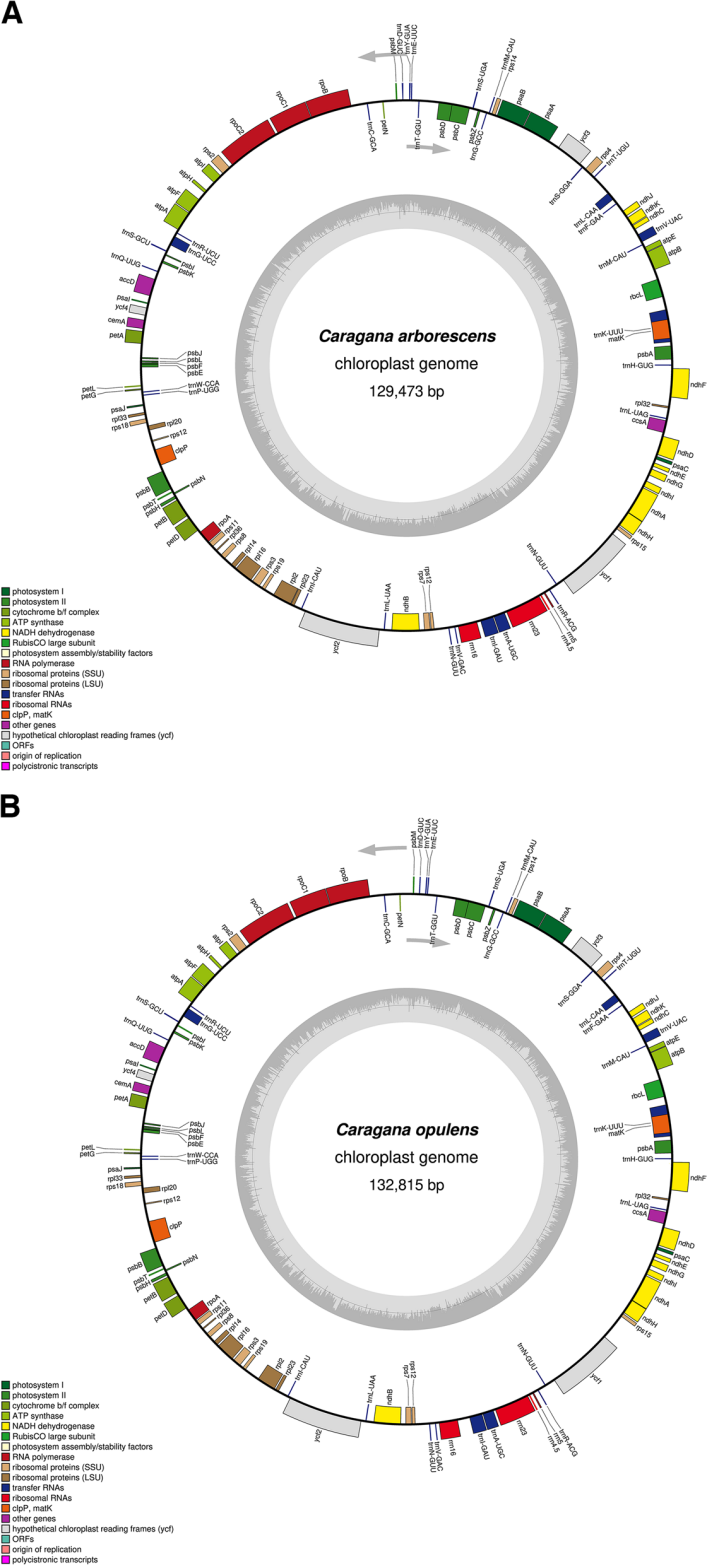
Chloroplast gene maps of *C. arborescens ***A** and *C. opulens ***B**. The genes within the circle are transcribed in a clockwise direction, and the genes outside the circle are transcribed in the opposite direction. Different color codes are used to depict functionally distinct genomes. In addition, the GC content in the inner ring is shown in light gray, whereas the change in the AT content is shown in dark gray

*C. arborescens* and *C. opulens* chloroplast genomes were sequenced using the Illumina NovaSeq platform. The whole genome sequence assemblies were 129,473 bp (Fig. 1A) and 132,815 bp (Fig. 1B). Due to the loss of the IR region, neither of the chloroplast genomes had the typical tetrad structure of most angiosperm chloroplast genomes, and their length was shortened accordingly. Nonetheless, the genetic structures were highly similar. In addition, we performed quality control on the sequencing data, and the Q20 and Q30 of *C. arborescens* and *C. opulens* were both greater than 90% (Additional file 1). To ensure the correctness of the assembly, we carried out quality control on the assembly results and verified that the two *Caragana* plants lacked the IR region. (Additional files 2 and 3)

In the chloroplast genomes of *C. arborescens* and *C. opulens*, there were 111 unique genes, including 76 protein-coding genes, 31 tRNA genes, and 4 rRNA genes. The GC contents of *C. arborescens* and *C. opulens* were very similar, with values of 34.30% and 34.71%, respectively (Table 1). Four chloroplast genomes in the *Caragana* genus (*C. rosea, C. microphylla, C. kozlowii*, and *C. korshinskii*) with missing IR regions were compared with *C. arborescens* and *C. opulens* chloroplast genomes. The total sequence length varied between 129,331 and 133,122 bp. Due to the absence of the IR region, the chloroplast genome length of *C. korshinskii* was the shortest, at only 129,311 bp, and that of *C. rosea* was the longest, at 133,122 bp. In addition, there was one more gene in *C. arborescens* and *C. opulens* than in other species (tRNA encoded by the *trnN-GUU* gene), whereas the numbers of protein-coding genes and rRNA genes were consistent among the six plants. *C. rosea* had the highest GC content in its chloroplast genome, at 34.84% (%), followed by *C. kozlowii* (34.5%), and *C. microphylla* had the lowest GC content (34.2%). We also examined variation in the GC content among the three gene types. The GC content was stable and highest in rRNA (over 50%), followed by tRNA, and the GC content of protein-coding genes was approximately 37%. In conclusion, the sequence lengths and gene numbers of the chloroplast genomes of the six *Caragana* species were generally similar, and the average GC content of the species was approximately 34%, suggesting that the genomes in *Caragana* were relatively conserved.

**Table 1:** Summary of complete chloroplast genomes for six *Caragana* species

Plastome Characteristics		Caragana arborescens	Caragana opulens	Caragana kozlowii	Caragana rosea	Caragana microphylla	Caragana korshinskii
Protein-coding genes	Length (bp)	66,222	66,333	66,234	66,243	66,231	66,231
	GC (%)	36.89	37.01	37.03	37.13	36.88	36.88
	Length (%)	51.15	50.0	50.45	49.76	50.94	51.21
	Number	76	76	76	76	76	76
tRNA	Length (bp)	2,379	2,370	2,285	2,359	2,370	2,379
	GC (%)	52.74	52.83	53.15	52.73	53.14	53.05
	Length (%)	1.83	1.80	1.74	1.77	1.82	1.83
	Number	31	31	30	30	30	30
rRNA	Length (bp)	4,522	4,520	4,521	4,537	4,520	4,520
	GC (%)	54.8	54.56	54.75	54.77	54.82	54.82
	Length (%)	3.49	3.40	3.44	3.4	3.48	3.49
	Number	4	4	4	4	4	4
Total	Length (bp)	129,473	132,815	131,274	133,122	130,029	129,331
	Number Of genes	111	111	110	110	110	110
	GC (%)	34.3	34.71	34.5	34.84	34.26	34.36

Comparable to the genomes of other species, the chloroplast genomes of *C. arborescens* and *C. opulens* encode three categories of genes (Table 2). There were 57 genes associated with self-replication, encompassing ribosomal RNA, transfer RNA, and three subunits (large, small, and DNA-dependent RNA polymerase) responsible for encoding chloroplast RNA polymerase. Additionally, there were 44 genes linked to photosynthesis, while the remaining genes were classified as other or unknown genes. In the chloroplast genomes of *C. arborescens* and *C. opulens*, 16 genes with introns were detected, one of which, *ycf3*, had two introns, and the remaining 15 genes (*trnK-UUU*, *trnV-UAC*, *trnL-CAA*, *rpoC1*, *atpF*, *trnG-UCC*, *clpP*, *petB*, *petD*, *rpl16*, *rpl2*, *ndhB*, *trnI-GAU*, *trnA-UGC*, and *ndhA*) had only one intron (Additional file 4). Among these 16 intron-containing genes, the intron lengths in the two species were remarkably similar.

**Table 2:** Genes in the chloroplast genome of *Caragana* species

Category	Group of genes	Name of genes
Self-replication	Proteins of large ribosomal subunit	*rpl14*, *rpl16*^a^, *rpl2*^a^, *rpl20*, *rpl23*, *rpl32*, *rpl33*, *rpl36*
	Proteins of small ribosomal subunit	*rps11*, *rps12*^a^, *rps14*, *rps15*, *rps18*, *rps19*, *rps2*, *rps3*, *rps4*, *rps7*, *rps8*
	Subunits of RNA polymerase	*rpoA*, *rpoB*, *rpoC1*^a^, *rpoC2*
	Ribosomal RNAs	*rrn16*, *rrn23*, *rrn4.5*, *rrn5*
	Transfer RNAs	*trnA-UGC*^a^, *trnC-GCA*, *trnD-GUC*, *trnE-UUC*, *trnF-GAA*, *trnG-GCC*, *trnG-UCC*^a^, *trnH-GUG*, *trnI-CAU*, *trnI-GAU*^a^, *trnK-UUU*^a^, *trnL-CAA*^a^, *trnL-UAA*, *trnL-UAG*, *trnM-CAU*, *trnN-GUU*(2), *trnP-UGG*, *trnQ-UUG*, *trnR-ACG*, *trnR-UCU*, *trnS-GCU*, *trnS-GGA*, *trnS-UGA*, *trnT-GGU*, *trnT-UGU*, *trnV-GAC*, *trnV-UAC*^a^, *trnW-CCA*, *trnY-GUA*, *trnfM-CAU*
Photosynthesis	Subunits of photosystem I	*psaA*, *psaB*, *psaC*, *psaI*, *psaJ*
	Subunits of photosystem II	*psbA*, *psbB*, *psbC*, *psbD*, *psbE*, *psbF*, *psbH*, *psbI*, *psbJ*, *psbK*, *psbL*, *psbM*, *psbN*, *psbT*, *psbZ*
	Subunits of NADH dehydrogenase	*ndhA*^a^, *ndhB*^a^, *ndhC*, *ndhD*, *ndhE*, *ndhF*, *ndhG*, *ndhH*, *ndhI*, *ndhJ*, *ndhK*
	Subunits of cytochrome b/f complex	*petA*, *petB*^a^, *petD*^a^, *petG*, *petL*, *petN*
	Subunits of ATP synthase	*atpA*, *atpB*, *atpE*, *atpF*^a^, *atpH*, *atpI*
	Large subunit of rubisco	*rbcL*
Other genes	Maturase	*matK*
	Protease	*clpP*
	Envelope membrane protein	*cemA*
	Acetyl-CoA carboxylase	*accD*
	c-type cytochrome synthesis gene	*ccsA*
Unknown	Conserved hypothetical chloroplast ORF	*ycf1*, *ycf2*, *ycf3*^b^, *ycf4*

Gene^a^: Gene with one introns; Gene^b^: Gene with two introns; Gene(2): Number of copies of multi-copy genes

### Analyses of repetitive sequences and SSRs

Repeat sites are important in genomic evolution, such as in structural rearrangement and size-based evolution [32, 33]. In this study, we identified repetitive sequences in the chloroplast genomes of *C. arborescens* and *C. opulens* and analyzed their content. The chloroplast genome with a repeat length greater than or equal to 30 bp contained four types of repeats: forward (F), palindromic (P), reverse (R), and complementary (C) repeats. In the two plants, 129 (length range: 30–249 bp) and 229 (length range: 30–472 bp) repeats, respectively, were identified (Additional file 5). The length range of 30–49 bp had the highest frequency among all classes of repetitive sequences (*C. arborescens*: 68.22%, *C. opulens*: 52.40%).

Structural analysis of the repetitive sequences showed that the *C. arborescens* genome was composed of 85 forward repeats (65.89%), 36 palindromic repeats (27.91%), 7 reverse repeats (5.43%), and 1 complementary repeat (0.78%) (Additional file 6, Fig. 2). There were no complementary repeats in the *C. opulens* genome, which consisted of 165 forward repeats (72.05%), 62 palindromic repeats (27.07%), and 2 reverse repeats (0.87%) (Additional file 7, Fig. 2). The majority of repeat sequences existed in the intergenic spacer (IGS) region, and the majority were forward repeats.

**Fig. 2:**
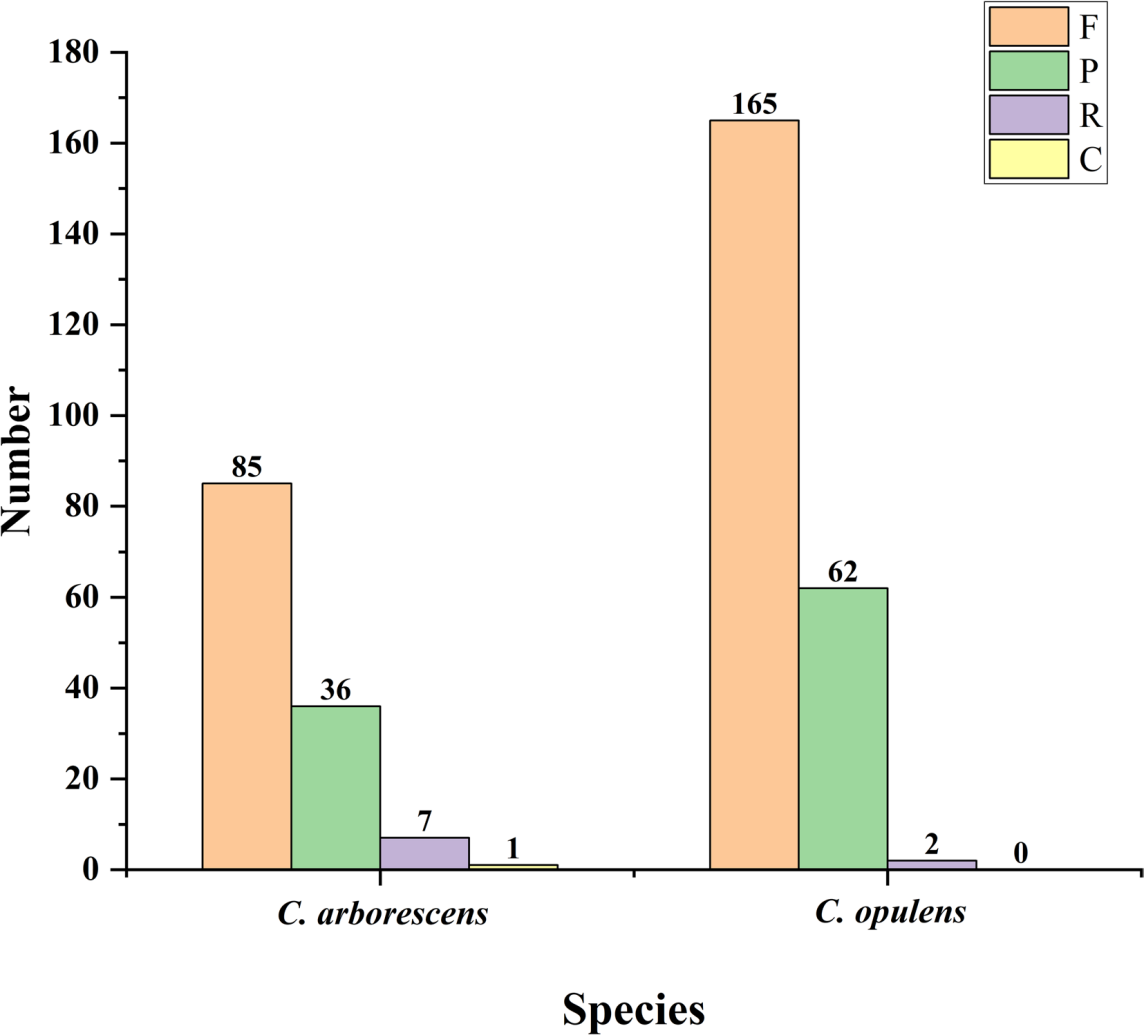
Repetitive sequences in the chloroplast genomes of *C. arborescens* and *C. opulens*

Plant chloroplast genomes harbor numerous simple sequence repeats (SSRs). This form of sequence is transmitted from parents to offspring. It has a relatively basic structure and low variability. SSRs are therefore efficient molecular markers [34]. Using MISA v1.0, we identified 18 types in the two *Caragana* species. The chloroplast genomes of *C. arborescens* and *C. opulens* contained 277 and 265 SSR loci, respectively (Additional file 8). The proportion of SSRs classified as mononucleotides in the two *Caragana* plants was highest, with estimates of 57.04% and 63.40%, respectively. Dinucleotide and trinucleotide repeat sequences accounted for 7.58% and 29.24% in the former species, and tetranucleotide repeat sequences accounted for the smallest proportion (6.14%). In the latter, the proportions of dinucleotide, trinucleotide, and tetranucleotide repeat sequences were 4.91%, 28.68%, and 2.64%, respectively, while pentanucleotide repeats represented the smallest proportion, 0.38%.

**Fig. 3:**
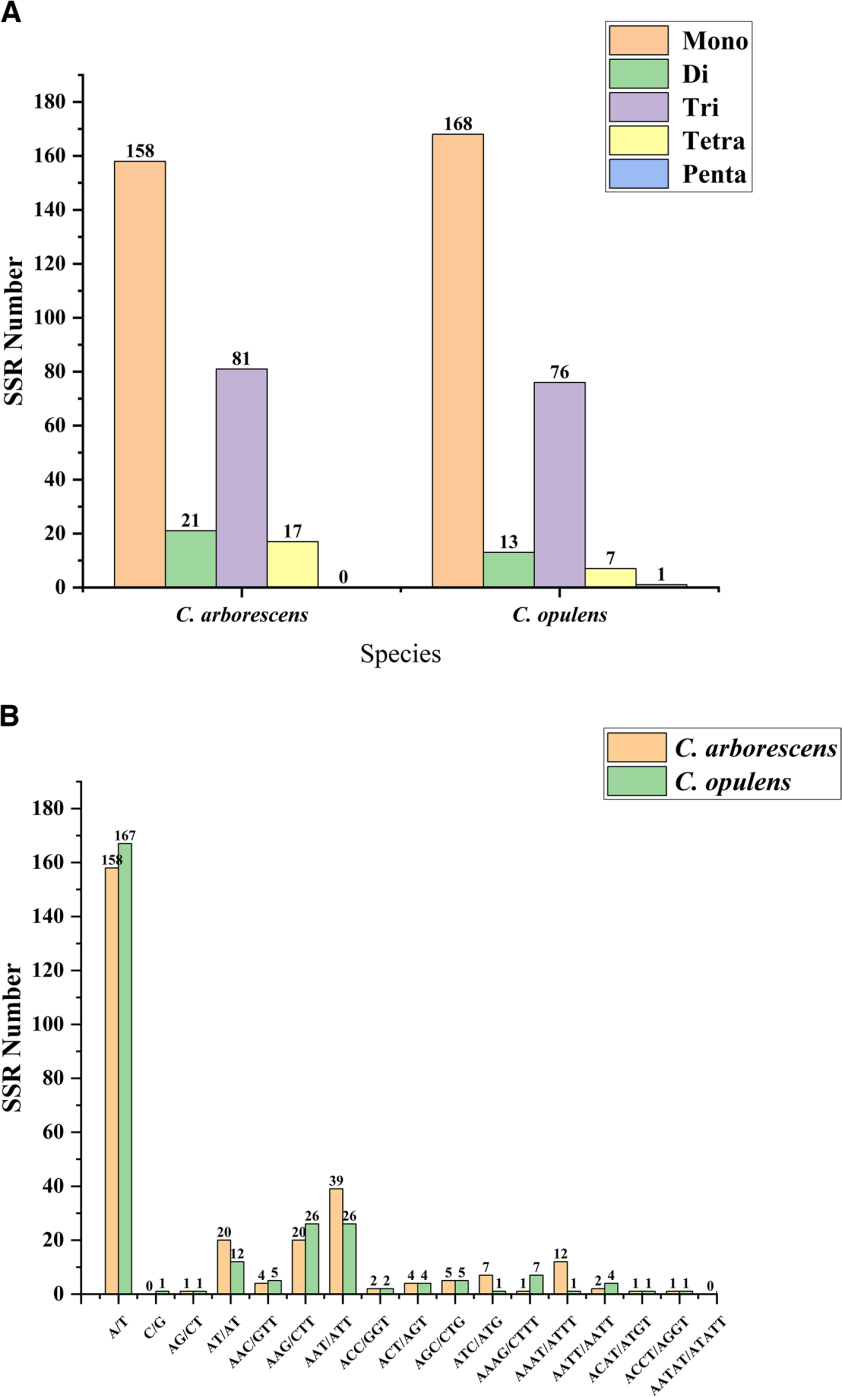
Simple sequence repeats (SSRs) identified in the plastid genomes of two *Caragana* species.  **A** Number of different SSR types found in the two genomes; **B** Number of SSRs with different repetition types

In *C. arborescens*, the longest SSR was detected in the *ycf1* gene of the chloroplast genome, which was a single nucleotide repeat sequence (A) with a length of 46 bp, whereas the longest SSR in *C. opulens* was a mononucleotide (T) with a length of 26 bp (Additional file 9). In addition, the distribution of SSRs in coding and noncoding regions was analyzed. Additional file 10 displays that the number of SSRs in the protein-coding region was significantly lower than that in the non-coding region. The majority of these SSRs were A/T single nucleotide repeats; 158 and 167 of the two *Caragana* species contained A/T, while only one contained C/G (Fig. 3A and B). Similarly, the majority of dinucleotide repeats consisted of AT/AT, resulting in a deviation in base composition, consistent with the finding that the overall AT content of plastids is greater than the GC content [35].

### Codon usage bias analysis

Plastids exhibit a prevalent codon usage bias. Codon usage bias may influence phylogenetic relationships and the molecular evolution of genes [36]; it can be used to study the origin, mutation model, and evolution of species. We analyzed the codon distribution in all protein-coding genes in these two plant species. The 76 protein-coding gene sequences of the two *Caragana* species were used to generate 44,033 codons in total. Leucine (Leu) was the amino acid with the highest content, accounting for 10.58% and 10.65%, respectively, followed by codons encoding isoleucine (Ile) (9% and 8.89%), while cysteine (Cys) had the lowest abundance in the two plants (Additional file 11).

**Fig. 4:**
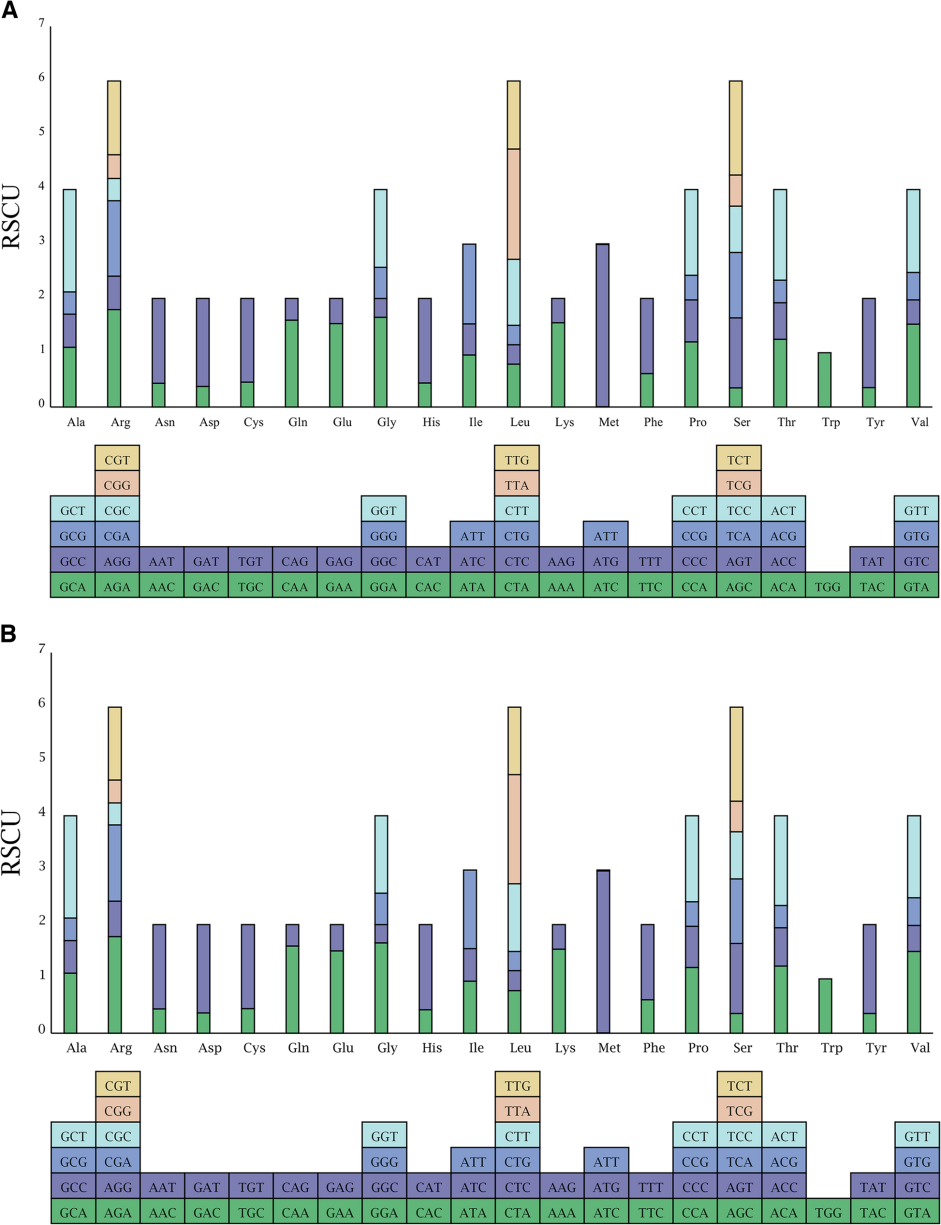
Amino acid frequencies of the chloroplast genomes of *C. arborescens ***A** and *C. opulens ***B**. The squares below represent all of the codons that encode each type of amino acid; the height of the column above represents the sum of RSCU values for all codons; the height of each column represents the RSCU value for each codon

We also independently calculated the relative synonymous codon usage (RSCU) values to evaluate the codon usage bias in the chloroplast genomes of the two species (Fig. 4). When the RSCU value is greater than 1.0, the codon is considered optimal. Among the 31 codons with RSCU values greater than 1.0, the AUG codon encoding methionine had the highest utilization bias (*C. arborescens* RSCU: 2.99 (Fig. 4A), *C. opulens* RSCU: 2.98 (Fig. 4B)). Tryptophane had no codon usage bias among these 31 codons (only one codon). Except for UUG, which encodes leucine, and AUG, which encodes methionine, the remaining codons terminated in A (12) or U (16) (Additional file 11).

### Sequence divergence analysis

**Fig. 5:**
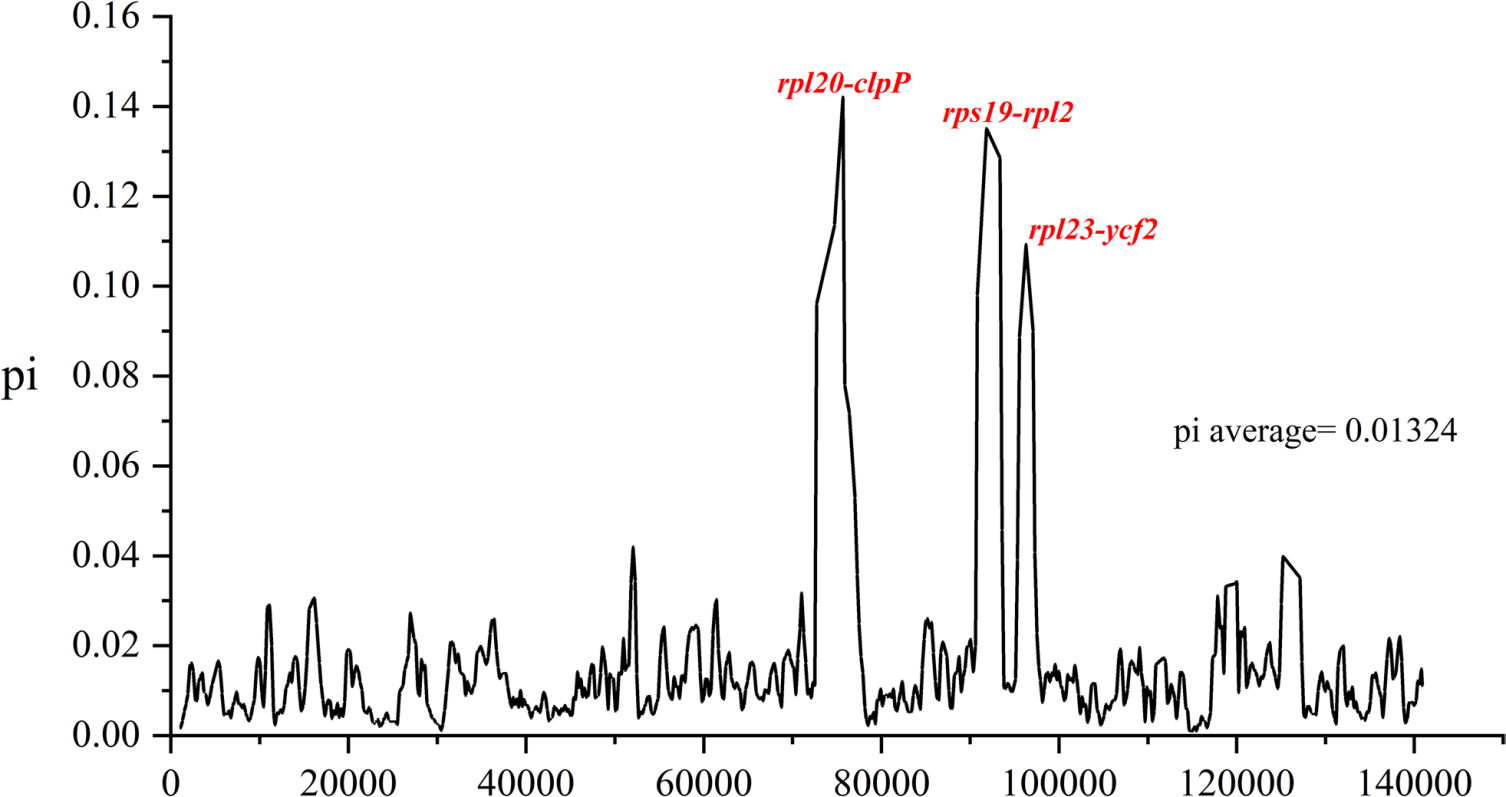
Nucleotide diversity (pi) for 111 genes in two *Caragana* species

Previous research has demonstrated that highly variable loci in the plastid genome can be used to develop molecular markers [13]. Therefore, DnaSP6 [37] was used to calculate nucleotide diversity (pi) in order to identify highly variable regions in the chloroplast genomes of six *Caragana* species. In a sliding window analysis, the Pi values of the six plants ranged from 0 to 0.14211, with an average value of approximately 0.01324 (Fig. 5), indicating that the chloroplast genome sequences within the genus had a high degree of similarity. *rpl20-clpP*, *rps19-rpl2*, and *rpl23-ycf2* were the most highly variable regions based on the pi values. Furthermore, the *rpl20-clpP* region had the highest nucleotide diversity (pi = 0.1438), followed by the *rpl23-ycf2* region (0.10584).

**Fig. 6:**
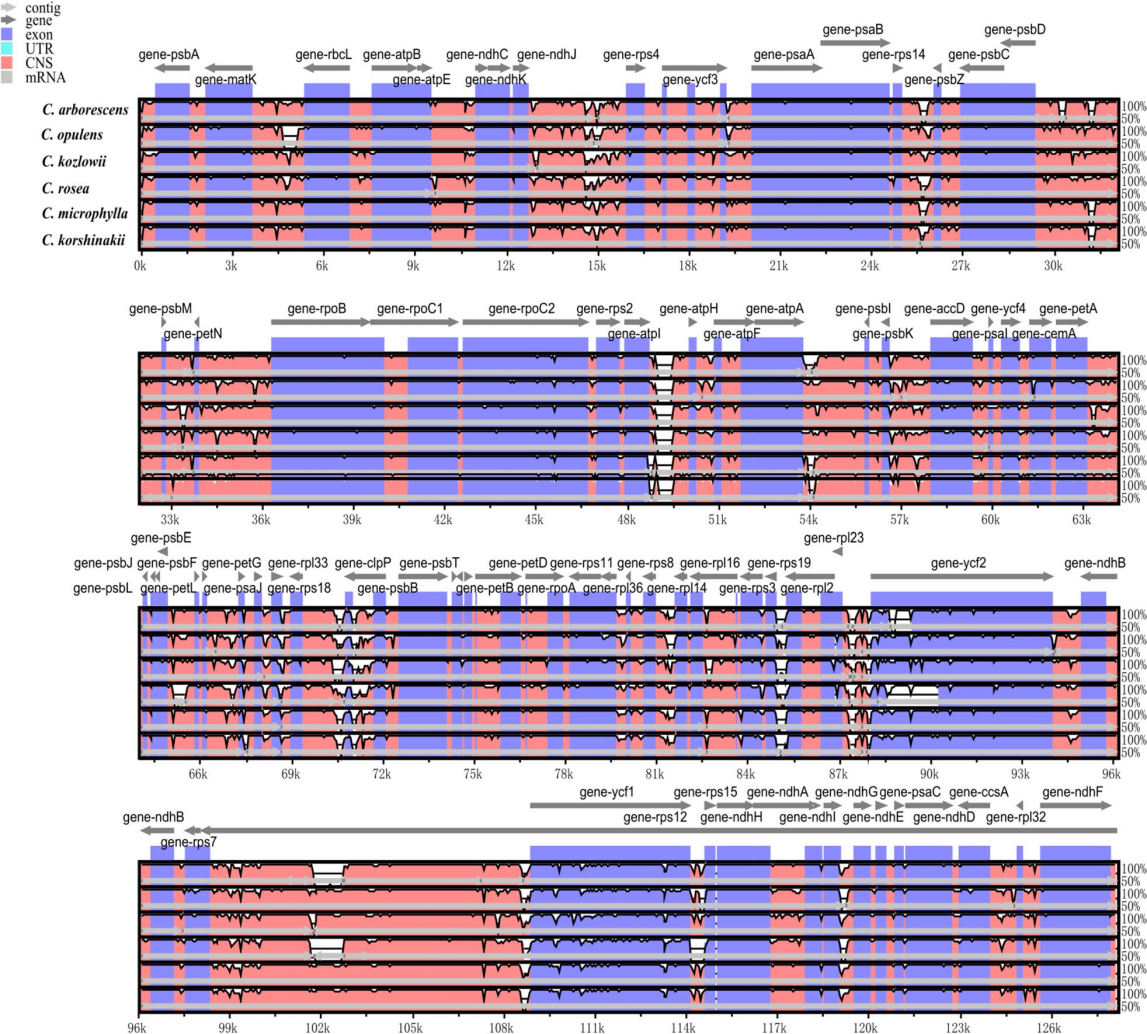
Chloroplast genome differences in six *Caragana* species, as determined using mVISTA. The gray arrow indicates the direction of translation. The *x*-axis represents the coordinates in the chloroplast genome. The *y*-axis represents the percentage between 50% and 100%. Blue indicates protein coding (exon); light green indicates untranslated region (UTR); orange indicates conserved non-coding sequences (CNSs).

Chloroplast genome sequence divergence was evaluated in *C. arborescens* and *C. opulens*. In particular, *C. arborescens*, *C. opulens*, *C. kozlowii*, *C. rosea*, *C. microphylla*, and *C. korshinskii* whole plastid genome sequences were compared with that of *C. jubata* (Fig. 6). Extremely low sequence divergence among species suggested that the chloroplast genome was conserved. IGS (*matK*-*rbcL*), IGS (*psbM*-*petN*), IGS (*atpA*-*psbI*), IGS (*petA*-*psbL*), IGS (*psbE*-*petL*), and IGS (*rps7*-*rps1 2*) exhibited significant differences among *Caragana* species. Additionally, the majority of protein-coding regions were highly conserved, with a few exceptions (*accD*, *ycf2*, and *rps7*). This indicates that IGS is responsible for the accelerated evolution of *Caragana* species.

### Phylogenetic analysis

To determine the phylogenetic position of *Caragana* in the family Fabaceae, we generated multiple sequence alignments based on 86 protein sequences commonly found in 23 plastids. In addition to *Caragana* sequences, additional sequences were obtained from *Wisteria* (1), *Glycyrrhiza* (2), *Astragalus* (1), *Calophaca* (1), *Cicer* (1), *Medicago* (3), *Trifolium* (3), and *Lathyrus* (4). Numbers in brackets indicate the number of species in the relevant group.

**Fig. 7:**
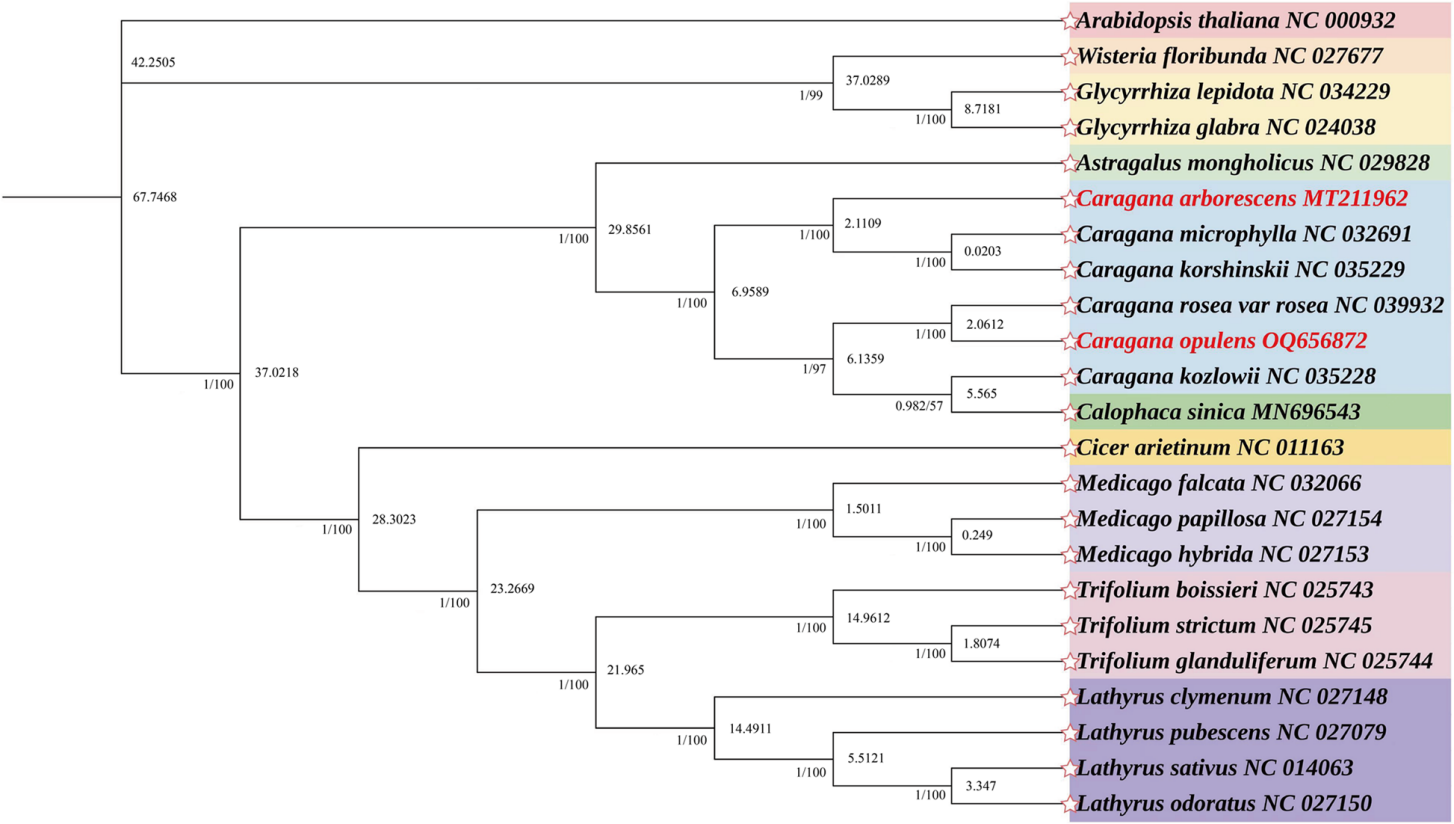
Phylogenetic tree based on 23 plant chloroplast genomes constructed using Bayesian inference and maximum likelihood. The number after the node represents the bootstrap support value. The GenBank accession numbers are shown after each species. *C. arborescens* and *C. opulens* are highlighted in red

Based on the chloroplast genomes of 22 Fabaceae and *Arabidopsis thaliana* (outgroup), phylogenetic trees were constructed by Bayesian and maximum likelihood methods. The phylogenetic trees acquired by the two methods showed a similar topology. Phylogenetic analysis revealed that all samples were classified into three main branches. The following two pairs showed a close relationship: *C. microphylla* and *C. korshinskii* as well as *C. opulens* and *C. rosea* (Fig. 7). Of note, the genera *Astragalus* and *Caragana* were relatively closely related (bootstrap support: 100%) and categorized into *Subtrib*. Astragalinae. This result was consistent with those of previous studies [2]. From Fig. 7, it can be seen that the divergence of *C. arborescens* from *C. opulens* occurred at about 6.9585 Mya.

## Discussion

*Caragana* is a superb forage native to northwest China and certain plateau regions, with significant value for the enhancement of natural pastures and the establishment of forage bases. *Caragana* plants are cultivated extensively owing to their resistance to drought, aridity, cold, and heat. Using the Illumina platform, we sequenced the complete chloroplast genomes of two *Caragana* species in this study. By assembling and annotating these genomes, more detailed information was obtained. Two plastids ranged in size from 129,473 to 132,815 bp and were found in *C. arborescens* and *C. opulens*, respectively. Other *Caragana* genomes have comparable gene structures. In some species, the chloroplast genome reportedly lacks *ycf2*, *rpl23*, and *accD* [38–40], whereas these genes were present in *Caragana*. Throughout plant evolution, several genes have been lost from the plastid genome. For example, the r*pl22* and *infA* genes were lost in some or all legumes [41], *infA* is an abnormally unstable flowering plant chloroplast gene, and *rpl22* encoding ribosomal protein *CL22* was lost in cpDNA and relocated to the nucleus [42, 43]. Similarly, in this study, the *infA* and *rpl22* loci were not found in *C. arborescens* and *C. opulens*. Recent research has demonstrated, however, that the *infA* gene is present in the chloroplast genomes of *C. jubata*, *C. erinacea*, and *C. bicolor* [2]. These results suggest that *infA* exists in some *Caragana* genomes.

As with the majority of plant species, the plastids of two *Caragana* species were conserved and no rearrangements were detected. Multiple *Caragana* species, such as *C. microphylla*, *C. erinacea*, and *C. intermedia*, have reportedly lost their IR region [2, 11, 12]. Similarly, the chloroplast genomes of *C. arborescens* and *C. opulens* examined in this study lacked the IR region, and the two plants shared a high degree of similarity in terms of genomic structure, gene deletion, genomic size, gene types, repeat sequence distribution, etc. Moreover, the cpDNA G/C content is a key determinant of inter-specific affinity [2], and the DNA G/C content of the two *Caragana* species evaluated in this study was highly similar. Numerous repetitive sequences were identified in the plastid genomes of two plants. These sequences are significant genetic markers and are closely associated with the origin and evolution of species [44]. Four types of repeats were identified in this study: complement repeats, reverse repeats, forward repeats, and palindromic repeats. The dispersed repeats were longer than 30 base pairs, and the repeat sequence length in the two *Caragana* species varied between 30 and 472 base pairs. In addition, SSRs are regarded as essential molecular markers for population genetic research and are widely employed to assess genetic diversity, phylogenetic relationships, and evolution [45]. In total, the chloroplast genomes of two *Caragana* species contained between 265 and 277 SSRs with a significant A/T bias. In this study, the majority of SSR types were single nucleotide repeats and non-coding regions (IGSs) contained the most SSRs. Similar results have been observed in other *Caragana* species, including *C. rosea*, *C. microphylla*, *C. korshinskii*, and *C. kozlowii* [27]. These repeat sequences provide a crucial starting point for the development of genetic indicators for *Caragana* species and can be utilized for phylogenetic and ecological research.

Codon utilization preference is related to the species of origin and the mutational model. The study of codon bias patterns in chloroplast genomes can shed light on plant phylogenetic relationships, gene expression mechanisms, and molecular evolution [36]. Leucine (Leu) is the most abundant amino acid in *C. arborescens* and *C. opulens* (mean of 1,969), and the same trend has been observed in other *Caragana* species. In addition, our research revealed that the majority of synonymous codons preferred based on RSCU values terminated in A/U, resulting in a high AT content in the gene. This may be explained by natural selection and gene mutation. Codon preference and utilization patterns reflect the evolutionary relationships between species to a limited extent [46]; however, additional research is required.

We identified five IGS with relatively high diversity (pi > 0.01037): *rpoC2*-*rps2*, *accD*-*cemA*, *rps18*-*clpP*, *rpoA*-*rpl36*, and *rpl2*-*rpl23*. In addition, fragments of the *ycf1*, *rps3*, and *rps7* genes exhibited comparatively high nucleotide diversity. These variable regions could potentially function as DNA barcode labels for phylogenetic relationships, species recognition, and population genetics research [47–49]. The sequence variation in six assembled *Caragana* plant genomes were then compared, revealing that the coding regions were more conserved than the untranslated regions, corroborating findings from other *Caragana* species.

A phylogenetic analysis of 23 Fabaceae species simultaneously revealed the phylogenetic position of *Caragana* and the relationships between *Caragana* and closely related species. The evolution of the plastid genome (nucleotide changes and structural changes) has been elucidated by phylogenetic methods [50, 51]. *Caragana* species were monophyletic, and *C. arborescens*, *C. opulens*, *C. kozlowii*, *C. rosea*, *C. microphylla*, and *C. korshinskii* were distinguishable from other species. Our findings may serve as a guide for future research on the evolution of *Caragana* and the creation of novel molecular markers. Our findings augment the chloroplast genome database for the genus *Caragana*.

## Conclusions

In this study, we assessed phylogenetic relationships based on the chloroplast genome sequences of *C. arborescens*, *C. opulens*, and 23 legume species belonging to the IRLC. The characterization of long repeats, SSRs, codon usage bias, and five hypervariable regions provides a basis for future work, such as the development of new molecular markers as well as population genetic and phylogenetic analyses. We analyzed the sequences and structures of the chloroplast genomes of two *Caragana* species as well as the evolutionary position and relationships with other species in the genus, providing a data basis for more in-depth and comprehensive studies aimed at *Caragana* species identification, analyses of genetic diversity, and phylogenetic research. Furthermore, our data expand the chloroplast genome database of *Caragana*.

## Materials and methods

### DNA extraction, library construction, and sequencing

*C. arborescens* and *C. opulens* leaves were collected in Qinghai Province (China) at the following coordinates: *C. arborescens*: 36° 43′ 24.80′′ N, 101° 44′ 54.11′′ E; *C. opulens*: 37° 36′ 53.34′′ N, 101° 19′ 18.63′′ E. An improved cetyltrimethylammonium bromide (CTAB) method was used to extract whole genome DNA from fresh leaves of *Caragana* plants [52]. Both the concentration and integrity of genomic DNA were measured via 1% agarose gel electrophoresis and Qubit 3.0 Fluorometer (Invitrogen, Ghent, Belgium). When the test was successful, the mechanical damage method was utilized to ultrasonically fragment the sample DNA, purify the genomic DNA, and stop the repair process. The DNA fragment size was subsequently determined using agarose gel electrophoresis, and the sequencing library was generated using PCR amplification. The qualified library was sequenced utilizing the Illumina NovaSeq platform to generate 150 bp pair-end reads.

### Gene annotation and sequence analyses

Trimmomatic v0.39 [53] was used to remove low-quality reads from the original data. The chloroplast genome sequence was then assembled using SPAdes v3.10.1 (http://cab.spbu.ru/software/spades/) [54] to obtain its seed sequence and for a K-mer analysis of the seed sequences to obtain contigs. SSPACE v2 [55] was used to connect the contigs and obtain the scaffold sequences. GapFiller v2.1.1 [56] was used to supplement the gaps found in the scaffold sequence to assure the integrity of the pseudogenome sequence. After adjusting the corrected pseudogenome sequence, complete chloroplast genome sequences of *C. arborescens* and *C. opulens* were obtained. We use software Bowtie2 to align sequencing reads to the genome, and then use SAMtools to calculate genome coverage and insert size distribution. Blast (https://blast.ncbi.nlm.nih.gov/Blast.cgi) was used to derive the chloroplast genome annotation results for the two plants. Hmmer (http://www.hmmer.org) and ARAGORN [57] (http://ogdraw.mpimp-golm.mpg.de/index.shtml) were used to obtain the rRNA and tRNA annotation information, respectively. OGDRAW [58] (http://ogdraw.mpimp-gol.m.mpg.de/index.html) was used to plot the chloroplast genome maps for *C. arborescens* and *C. opulens*. MT211962 and OQ656872 are the NCBI accession numbers for the newly obtained whole chloroplast genome data.

### Repeat structure, SSRs, and codon usage analysis

Vmatch Web [59] was used to identify repeats (forward repeats, palindromic repeats, reverse repeats, and complementary repeats). MISA [60] was used to identify SSRs in two *Caragana* species with the following search parameters: mononucleotides set to ≥ 10 repeat units, dinucleotides ≥ 8 repeat units, trinucleotides, tetranucleotides, pentanucleotides and hexanucleotides ≥ 3 repeat units. CodonW v1.4.2 was used to calculate the relative synonymous codon usage (RSCU) values of protein-coding genes using the default settings.

### Comparative genome analysis

*C. arborescens* and *C. opulens* whole chloroplast genome sequences were compared with those of *C. kozlowii*, *C. rosea*, *C. microphylla*, and *C. korshinskii* using mVISTA (Shuffle-LAGAN mode). As a reference, the *C. jubata* plastid was labeled. In total, 111 gene sequences in *C. arborescens* and *C. opulens* were aligned using MEGA7 [61]. To calculate the nucleotide diversity (Pi) values using DnaSP6, the following parameter configurations were utilized: Normal parameters, 200 bp step size and 600 bp window length [62].

### Phylogenetic analysis

A phylogenic tree was established using plastome sequences of 20 species (pertaining to IRLC) downloaded from NCBI database and the two species sequenced in this study, with *Arabidopsis thaliana* as an outgroup. All 23 complete chloroplast genomes were aligned using MAFFT (default parameters), and the aligned sequences were optimized using MACSE. Bayesian inference (BI) and maximum likelihood (ML) methods were used to construct phylogenetic trees. MrBayes was used for BI analysis. The substitution model was selected using ModelFinder [63] and was set to GTR + F + I + G4. The MrBayes analysis was set to run for 1,000 cycles, and the first 25% of cycles were removed as burn-in. The average standard deviation of splitfrequencies was set to > 0.01 [64]. IQ-TREE was used for ML analysis, with the automatic partitioning module and bootstrap analysis set to 1,000 repetitions to evaluate branch support. In addition, we also calculated the divergence time of *C. arborescens* and *C. opulens*. Two time standard points were found through Timetree5 [65]: the estimated divergence time between *Wisteria floribunda* and *Glycyrrhiza lepidota* is at least 37 Mya, the estimated divergence time between *Glycyrrhiza lepidota* and *Glycyrrhiza glabra* is at least 8 Mya. In BEAST v1.8.4 [66], the random local clock and the Yule speciation prior set were used to estimate the divergence time. The posterior distributions of parameters were obtained using MCMC analysis for 10 million generations with a burn-in percentage of 10%. FigTree v1.4.2 [67] was used to visualize the resulting tree and to obtain the divergence time.

### Supplementary Information

**Additional file 1: Table S1. **Statistical table of sequencing data.

**Additional file 2: Fig. S1. **Genome coverage of chloroplast genome assembly sequence of *Caragana arborescens.*

**Additional file 3: Fig. S2.** Genome coverage of chloroplast genome assembly sequence of *Caragana opulens.*

**Additional file 4: Table S2. **The intron-containing genes and the length of exons and introns in the chloroplast genomes of two *Caragana* species.

**Additional file 5: Table S3. **Types and numbers of Repeats in chloroplast genome of *C. arborescens *and *C.opulens.*

**Additional file 6: Fig. S3. **Numbers of four types of repeats found in *C. arborescens.*

**Additional file 7: Fig. S4. **Numbers of four types of repeats found in *C. opulens.*

**Additional file 8: Table S4. **Types and numbers of SSR in chloroplast genome of *C. arborescens *and *C.opulens.*

**Additional file 9: Table S5. **Distribution of SSRs in cp genome of *C. arborescens *and *C.opulens*.

**Additional file 10: Fig. S5. **The number of SSRs was found in coding (CDS), and intronic regions, intergenic (IGS), Respectively.

**Additional file 11: Table S6.  **Analysis of coding ability and codon preference of chloroplast genome.

## Data Availability

The original sequencing data have been submitted to the NCBI database and received GenBank accession numbers MT211962 (*C. arborescens*), OQ656872 (*C. opulens*). The data used in this study are available in the public domain (https://www.ncbi.nlm.nih.gov).
