# Supplementary material for: Chloroplast genome analyses of Caragana arborescens and Caragana opulens
Source: BMC Genom Data. 2024 Feb 9;25:16. doi: 10.1186/s12863-024-01202-4 (PMC10854190; doi:10.1186/s12863-024-01202-4)
Supplement: Supplementary file 1 — Additional file 1: Table S1. Statistical table of sequencing data. [file 12863_2024_1202_MOESM1_ESM.doc]

Table S1 Statistical table of sequencing data

| Sample ID | ReadSum | BaseSum | GC(%) | Q20(%) | Q30(%) |
| --- | --- | --- | --- | --- | --- |
| Caragana arborescens | 22855947 | 6856784100 | 38.31 | 96.89 | 91.69 |
| Caragana opulens | 21130518 | 6339155400 | 38.11 | 97.12 | 92.08 |
