# Supplementary material for: Chloroplast genome analyses of Caragana arborescens and Caragana opulens
Source: BMC Genom Data. 2024 Feb 9;25:16. doi: 10.1186/s12863-024-01202-4 (PMC10854190; doi:10.1186/s12863-024-01202-4)
Supplement: Supplementary file 2 — Additional file 2: Fig. S1. Genome coverage of chloroplast genome assembly sequence of Caragana arborescens. [file 12863_2024_1202_MOESM2_ESM.docx]

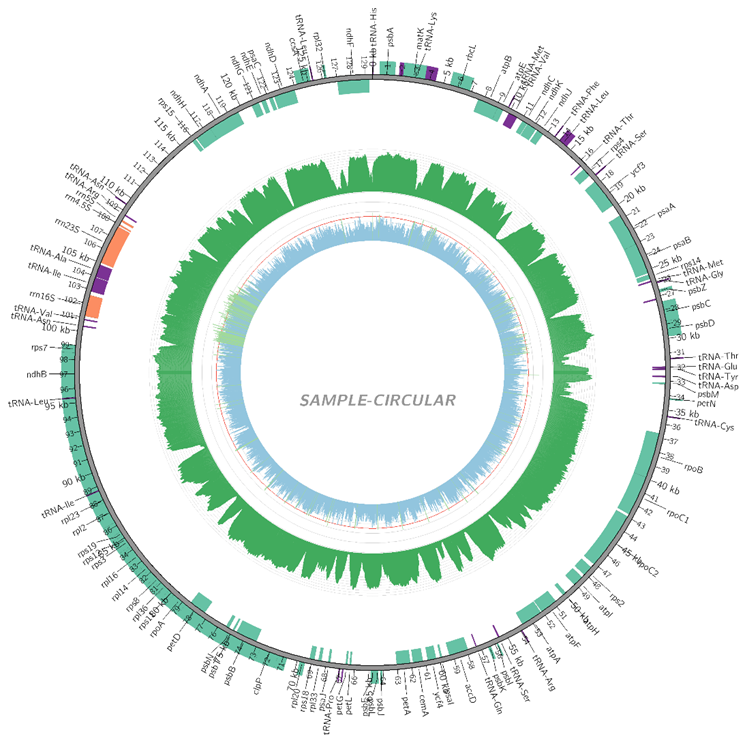


**Fig. S1** Genome coverage of chloroplast genome assembly sequence of *Caragana arborescens*

Note : The outermost circle represents the genome sequence ; the coding gene is represented by a green box ; tRNA is represented by purple box ; rRNA is represented by orange box ; the green ring inside represents the depth of coverage, the depth of the IR region is generally twice that of other regions; the innermost circle represents the GC content of the genome. Regions with GC content greater than 50% are indicated by green lines, while those with GC content greater than 50% are indicated by blue lines.
