# Supplementary material for: Chloroplast genome analyses of Caragana arborescens and Caragana opulens
Source: BMC Genom Data. 2024 Feb 9;25:16. doi: 10.1186/s12863-024-01202-4 (PMC10854190; doi:10.1186/s12863-024-01202-4)
Supplement: Supplementary file 4 — Additional file 4: Table S2. The intron-containing genes and the length of exons and introns in the chloroplast genomes of two Caragana species. [file 12863_2024_1202_MOESM4_ESM.doc]

Table S2 The intron-containing genes and the length of exons and introns in the chloroplast genomes of two *Caragana* species

| Species | Gene | Exon I(bp) | Intron I(bp) | Exon II(bp) | Intron II(bp) | Exon III(bp) |
| --- | --- | --- | --- | --- | --- | --- |
| *C.arborescens* | *trnK-UUU* | 37 | 2488 | 29 |  |  |
|  | *trnV-UAC* | 39 | 574 | 37 |  |  |
|  | *trnL-CAA* | 37 | 550 | 50 |  |  |
|  | *ycf3* | 126 | 713 | 228 | 877 | 153 |
|  | *rpoC1* | 432 | 789 | 1623 |  |  |
|  | *atpF* | 168 | 660 | 411 |  |  |
|  | *trnG-UCC* | 23 | 682 | 49 |  |  |
|  | *clpP* | 368 | 701 | 229 |  |  |
|  | *petB* | 6 | 818 | 642 |  |  |
|  | *petD* | 8 | 717 | 475 |  |  |
|  | *rpl16* | 9 | 1054 | 399 |  |  |
|  | *rpl2* | 396 | 685 | 435 |  |  |
|  | *ndhB* | 723 | 685 | 762 |  |  |
|  | *trnI-GAU* | 38 | 951 | 35 |  |  |
|  | *trnA-UGC* | 38 | 812 | 35 |  |  |
|  | *ndhA* | 552 | 1170 | 540 |  |  |
| *C.opulens* | *trnK-UUU* | 37 | 2485 | 29 |  |  |
|  | *trnV-UAC* | 39 | 574 | 37 |  |  |
|  | *trnL-CAA* | 37 | 534 | 50 |  |  |
|  | *ycf3* | 126 | 702 | 228 | 875 | 153 |
|  | *rpoC1* | 432 | 790 | 1623 |  |  |
|  | *atpF* | 168 | 679 | 411 |  |  |
|  | *trnG-UCC* | 23 | 682 | 49 |  |  |
|  | *clpP* | 368 | 1159 | 223 |  |  |
|  | *petB* | 6 | 826 | 642 |  |  |
|  | *petD* | 8 | 720 | 475 |  |  |
|  | *rpl16* | 9 | 1108 | 399 |  |  |
|  | *rpl2* | 396 | 692 | 435 |  |  |
|  | *ndhB* | 723 | 685 | 762 |  |  |
|  | *trnI-GAU* | 38 | 953 | 35 |  |  |
|  | *trnA-UGC* | 38 | 810 | 35 |  |  |
|  | *ndhA* | 552 | 1169 | 540 |  |  |
