# Supplementary material for: Chloroplast genome analyses of Caragana arborescens and Caragana opulens
Source: BMC Genom Data. 2024 Feb 9;25:16. doi: 10.1186/s12863-024-01202-4 (PMC10854190; doi:10.1186/s12863-024-01202-4)
Supplement: Supplementary file 5 — Additional file 5: Table S3. Types and numbers of Repeats in chloroplast genome of C. arborescens and C.opulens. [file 12863_2024_1202_MOESM5_ESM.doc]

Table S3 Types and numbers of Repeats in chloroplast genome of *C. arborescens* and *C.opulens*

| Length | *C.arborescens* | | | |  | *C.opulens* | | | |  |
| --- | --- | --- | --- | --- | --- | --- | --- | --- | --- | --- |
|  | F | P | R | C | Total | F | P | R | C | Total |
| 30 | 15 | 4 | 4 | 1 | 24 | 20 | 5 | 1 | 0 | 26 |
| 31 | 6 | 3 | 1 | 0 | 10 | 4 | 2 | 1 | 0 | 7 |
| 32 | 1 | 3 | 0 | 0 | 4 | 2 | 2 | 0 | 0 | 4 |
| 33 | 2 | 1 | 0 | 0 | 3 | 8 | 3 | 0 | 0 | 11 |
| 34 | 5 | 4 | 1 | 0 | 10 | 5 | 1 | 0 | 0 | 6 |
| 35 | 1 | 1 | 0 | 0 | 2 | 5 | 6 | 0 | 0 | 11 |
| 36 | 0 | 1 | 0 | 0 | 1 | 7 | 0 | 0 | 0 | 7 |
| 37 | 2 | 0 | 1 | 0 | 3 | 2 | 0 | 0 | 0 | 2 |
| 38 | 1 | 1 | 0 | 0 | 2 | 12 | 2 | 0 | 0 | 14 |
| 39 | 2 | 1 | 0 | 0 | 3 | 5 | 2 | 0 | 0 | 7 |
| 40 | 3 | 1 | 0 | 0 | 4 | 2 | 2 | 0 | 0 | 4 |
| 41 | 7 | 1 | 0 | 0 | 8 | 3 | 1 | 0 | 0 | 4 |
| 42 | 2 | 3 | 0 | 0 | 5 | 2 | 3 | 0 | 0 | 5 |
| 43 | 0 | 0 | 0 | 0 | 0 | 1 | 0 | 0 | 0 | 1 |
| 44 | 1 | 0 | 0 | 0 | 1 | 2 | 0 | 0 | 0 | 2 |
| 45 | 0 | 2 | 0 | 0 | 2 | 2 | 2 | 0 | 0 | 4 |
| 46 | 3 | 1 | 0 | 0 | 4 | 1 | 1 | 0 | 0 | 2 |
| 47 | 1 | 0 | 0 | 0 | 1 | 0 | 0 | 0 | 0 | 0 |
| 48 | 0 | 0 | 0 | 0 | 0 | 1 | 0 | 0 | 0 | 1 |
| 49 | 1 | 0 | 0 | 0 | 1 | 2 | 0 | 0 | 0 | 2 |
| 50 | 0 | 1 | 0 | 0 | 1 | 0 | 0 | 0 | 0 | 0 |
| 51 | 0 | 1 | 0 | 0 | 1 | 0 | 0 | 0 | 0 | 0 |
| 52 | 2 | 0 | 0 | 0 | 2 | 1 | 1 | 0 | 0 | 2 |
| 53 | 1 | 0 | 0 | 0 | 1 | 2 | 2 | 0 | 0 | 4 |
| 54 | 2 | 0 | 0 | 0 | 2 | 1 | 0 | 0 | 0 | 1 |
| 55 | 0 | 0 | 0 | 0 | 0 | 0 | 1 | 0 | 0 | 1 |
| 56 | 1 | 0 | 0 | 0 | 1 | 1 | 0 | 0 | 0 | 1 |
| 57 | 3 | 2 | 0 | 0 | 5 | 1 | 0 | 0 | 0 | 1 |
| 58 | 0 | 0 | 0 | 0 | 0 | 7 | 0 | 0 | 0 | 7 |
| 59 | 0 | 0 | 0 | 0 | 0 | 1 | 1 | 0 | 0 | 2 |
| 60 | 1 | 0 | 0 | 0 | 1 | 0 | 1 | 0 | 0 | 1 |
| 61 | 1 | 2 | 0 | 0 | 3 | 5 | 0 | 0 | 0 | 5 |
| 62 | 1 | 0 | 0 | 0 | 1 | 2 | 2 | 0 | 0 | 4 |
| 63 | 2 | 0 | 0 | 0 | 2 | 5 | 2 | 0 | 0 | 7 |
| 64 | 1 | 0 | 0 | 0 | 1 | 1 | 0 | 0 | 0 | 1 |
| 66 | 0 | 0 | 0 | 0 | 0 | 1 | 0 | 0 | 0 | 1 |
| 67 | 0 | 0 | 0 | 0 | 0 | 2 | 0 | 0 | 0 | 2 |
| 69 | 1 | 0 | 0 | 0 | 1 | 0 | 0 | 0 | 0 | 0 |
| 70 | 0 | 0 | 0 | 0 | 0 | 1 | 1 | 0 | 0 | 2 |
| 71 | 0 | 1 | 0 | 0 | 1 | 0 | 0 | 0 | 0 | 0 |
| 73 | 0 | 0 | 0 | 0 | 0 | 2 | 0 | 0 | 0 | 2 |
| 74 | 0 | 0 | 0 | 0 | 0 | 1 | 2 | 0 | 0 | 3 |
| 75 | 0 | 1 | 0 | 0 | 1 | 3 | 2 | 0 | 0 | 5 |
| 76 | 0 | 0 | 0 | 0 | 0 | 3 | 0 | 0 | 0 | 3 |
| 78 | 1 | 0 | 0 | 0 | 1 | 1 | 0 | 0 | 0 | 1 |
| 80 | 1 | 0 | 0 | 0 | 1 | 0 | 0 | 0 | 0 | 0 |
| 81 | 2 | 0 | 0 | 0 | 2 | 1 | 0 | 0 | 0 | 1 |
| 82 | 0 | 0 | 0 | 0 | 0 | 1 | 0 | 0 | 0 | 1 |
| 83 | 0 | 0 | 0 | 0 | 0 | 1 | 1 | 0 | 0 | 2 |
| 84 | 0 | 0 | 0 | 0 | 0 | 1 | 0 | 0 | 0 | 1 |
| 85 | 2 | 0 | 0 | 0 | 2 | 2 | 1 | 0 | 0 | 3 |
| 86 | 1 | 0 | 0 | 0 | 1 | 0 | 0 | 0 | 0 | 0 |
| 88 | 0 | 0 | 0 | 0 | 0 | 1 | 0 | 0 | 0 | 1 |
| 91 | 0 | 0 | 0 | 0 | 0 | 1 | 0 | 0 | 0 | 1 |
| 92 | 1 | 0 | 0 | 0 | 1 | 0 | 0 | 0 | 0 | 0 |
| 93 | 1 | 0 | 0 | 0 | 1 | 1 | 0 | 0 | 0 | 1 |
| 95 | 0 | 0 | 0 | 0 | 0 | 1 | 0 | 0 | 0 | 1 |
| 96 | 0 | 0 | 0 | 0 | 0 | 1 | 0 | 0 | 0 | 1 |
| 100 | 1 | 0 | 0 | 0 | 1 | 2 | 1 | 0 | 0 | 3 |
| 102 | 1 | 0 | 0 | 0 | 1 | 0 | 0 | 0 | 0 | 0 |
| 106 | 1 | 0 | 0 | 0 | 1 | 0 | 0 | 0 | 0 | 0 |
| 107 | 0 | 1 | 0 | 0 | 1 | 0 | 1 | 0 | 0 | 1 |
| 110 | 0 | 0 | 0 | 0 | 0 | 2 | 1 | 0 | 0 | 3 |
| 116 | 1 | 0 | 0 | 0 | 1 | 0 | 0 | 0 | 0 | 0 |
| 117 | 0 | 0 | 0 | 0 | 0 | 1 | 0 | 0 | 0 | 1 |
| 118 | 0 | 0 | 0 | 0 | 0 | 1 | 0 | 0 | 0 | 1 |
| 124 | 0 | 0 | 0 | 0 | 0 | 1 | 1 | 0 | 0 | 2 |
| 127 | 0 | 0 | 0 | 0 | 0 | 1 | 0 | 0 | 0 | 1 |
| 130 | 1 | 0 | 0 | 0 | 1 | 0 | 0 | 0 | 0 | 0 |
| 131 | 0 | 0 | 0 | 0 | 0 | 1 | 0 | 0 | 0 | 1 |
| 132 | 0 | 0 | 0 | 0 | 0 | 1 | 0 | 0 | 0 | 1 |
| 134 | 0 | 0 | 0 | 0 | 0 | 1 | 0 | 0 | 0 | 1 |
| 135 | 0 | 0 | 0 | 0 | 0 | 1 | 0 | 0 | 0 | 1 |
| 153 | 0 | 0 | 0 | 0 | 0 | 0 | 1 | 0 | 0 | 1 |
| 154 | 1 | 0 | 0 | 0 | 1 | 0 | 0 | 0 | 0 | 0 |
| 160 | 0 | 0 | 0 | 0 | 0 | 1 | 0 | 0 | 0 | 1 |
| 175 | 0 | 0 | 0 | 0 | 0 | 1 | 0 | 0 | 0 | 1 |
| 178 | 0 | 0 | 0 | 0 | 0 | 1 | 1 | 0 | 0 | 2 |
| 195 | 0 | 0 | 0 | 0 | 0 | 0 | 1 | 0 | 0 | 1 |
| 202 | 0 | 0 | 0 | 0 | 0 | 1 | 0 | 0 | 0 | 1 |
| 213 | 0 | 0 | 0 | 0 | 0 | 0 | 1 | 0 | 0 | 1 |
| 215 | 0 | 0 | 0 | 0 | 0 | 1 | 0 | 0 | 0 | 1 |
| 221 | 0 | 0 | 0 | 0 | 0 | 0 | 1 | 0 | 0 | 1 |
| 240 | 0 | 0 | 0 | 0 | 0 | 1 | 0 | 0 | 0 | 1 |
| 249 | 1 | 0 | 0 | 0 | 1 | 0 | 0 | 0 | 0 | 0 |
| 252 | 0 | 0 | 0 | 0 | 0 | 1 | 0 | 0 | 0 | 1 |
| 255 | 0 | 0 | 0 | 0 | 0 | 0 | 1 | 0 | 0 | 1 |
| 262 | 0 | 0 | 0 | 0 | 0 | 1 | 0 | 0 | 0 | 1 |
| 264 | 0 | 0 | 0 | 0 | 0 | 1 | 0 | 0 | 0 | 1 |
| 305 | 0 | 0 | 0 | 0 | 0 | 2 | 0 | 0 | 0 | 2 |
| 328 | 0 | 0 | 0 | 0 | 0 | 1 | 0 | 0 | 0 | 1 |
| 396 | 0 | 0 | 0 | 0 | 0 | 0 | 1 | 0 | 0 | 1 |
| 419 | 0 | 0 | 0 | 0 | 0 | 1 | 0 | 0 | 0 | 1 |
| 429 | 0 | 0 | 0 | 0 | 0 | 1 | 0 | 0 | 0 | 1 |
| 443 | 0 | 0 | 0 | 0 | 0 | 0 | 1 | 0 | 0 | 1 |
| 444 | 0 | 0 | 0 | 0 | 0 | 0 | 1 | 0 | 0 | 1 |
| 472 | 0 | 0 | 0 | 0 | 0 | 1 | 0 | 0 | 0 | 1 |
| Total | 85 | 36 | 7 | 1 | 129 | 165 | 62 | 2 | 0 | 229 |
| propotion | 65.89% | 27.91% | 5.43% | 0.78% |  | 72.05% | 27.07% | 0.87% | 0 |  |
