# Supplementary figures and images for: Chloroplast genome analyses of Caragana arborescens and Caragana opulens
Source: BMC Genom Data. 2024 Feb 9;25:16. doi: 10.1186/s12863-024-01202-4 (PMC10854190; doi:10.1186/s12863-024-01202-4)

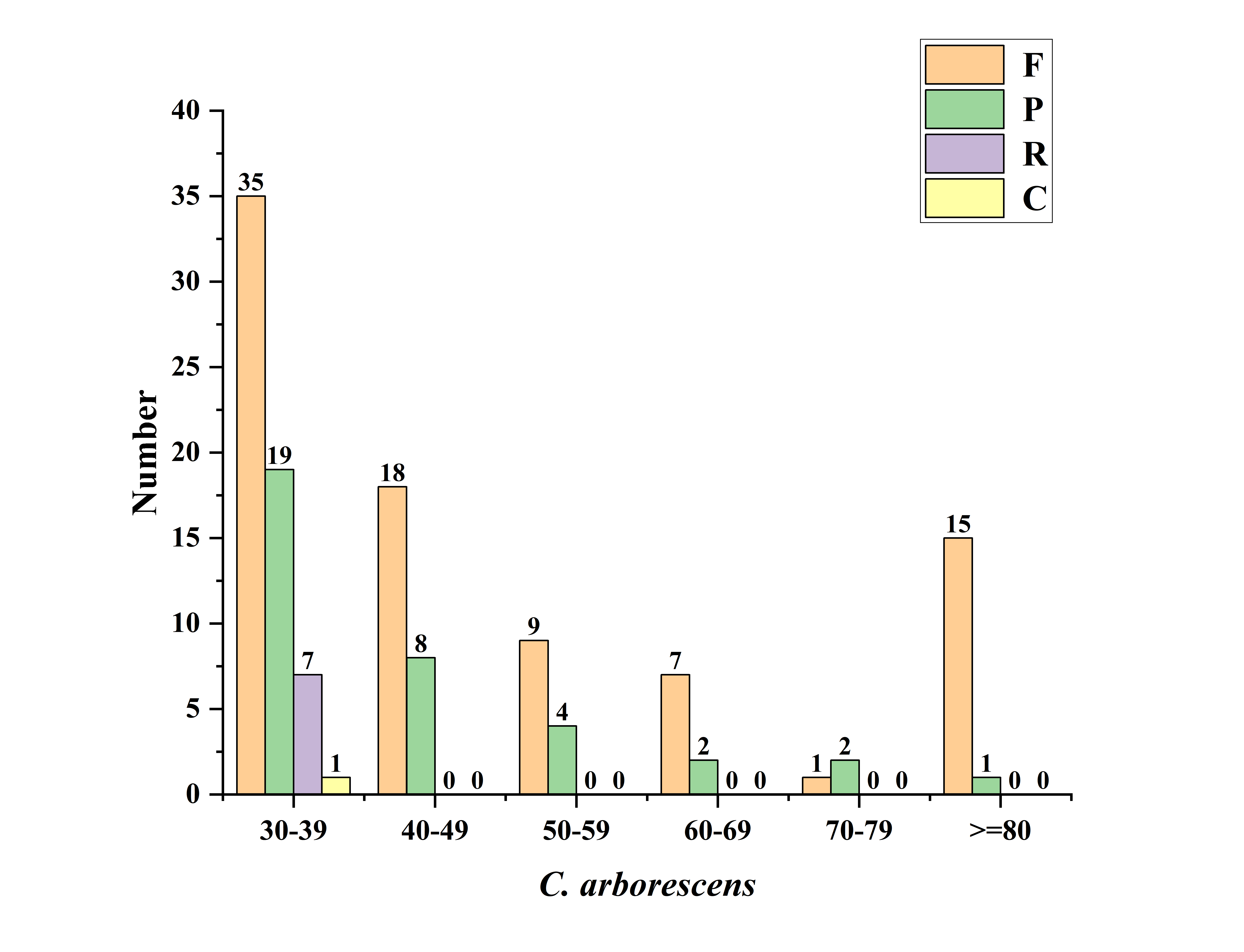


**Fig. S3** Numbers of four types of repeats found in *C. arborescens*

Supplement: Supplementary file 6 — Additional file 6: Fig. S3. Numbers of four types of repeats found in C. arborescens. [file 12863_2024_1202_MOESM6_ESM.doc]

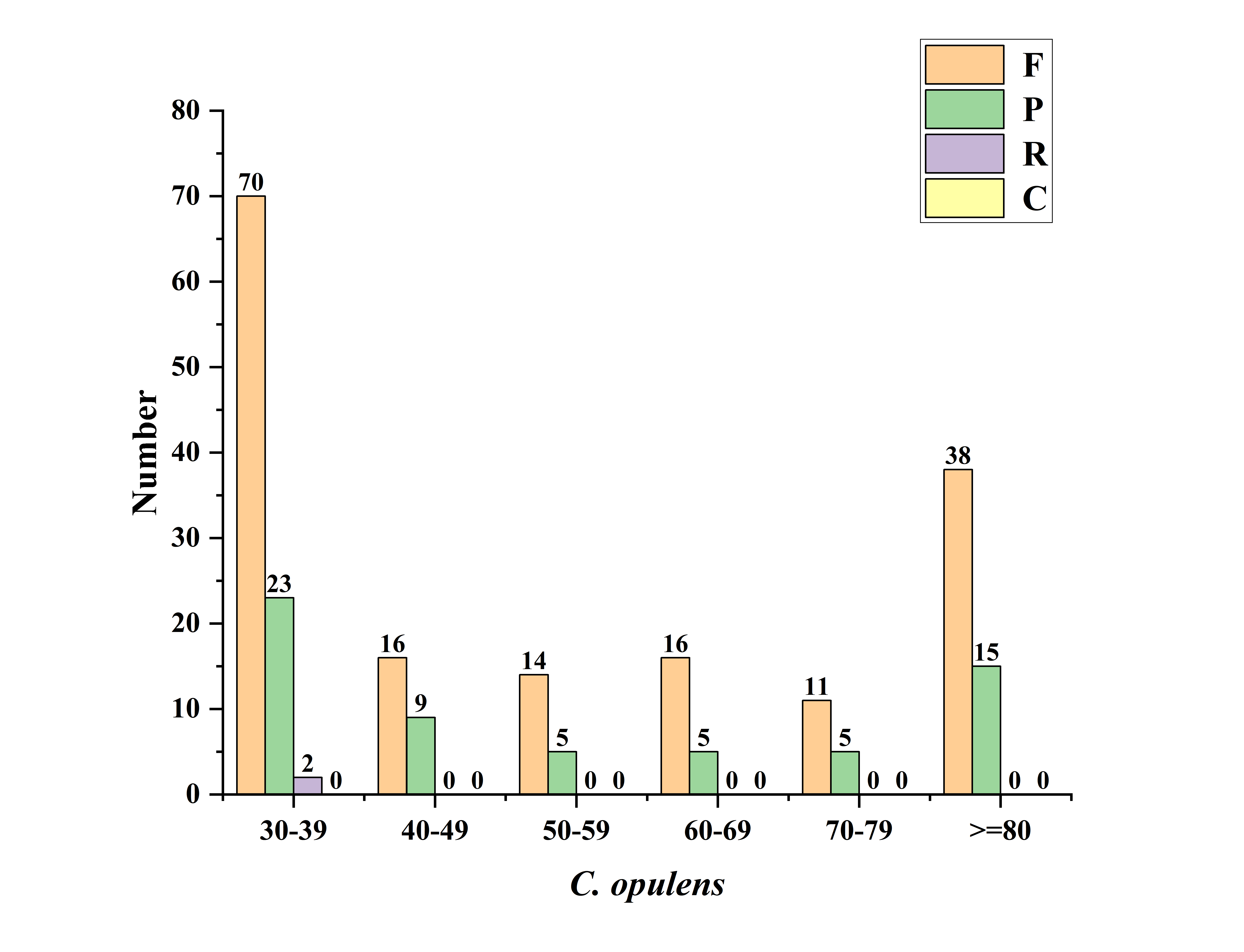


**Fig. S4** Numbers of four types of repeats found in *C. opulens*

Supplement: Supplementary file 7 — Additional file 7: Fig. S4. Numbers of four types of repeats found in C. opulens. [file 12863_2024_1202_MOESM7_ESM.doc]
