# Supplementary material for: Chloroplast genome analyses of Caragana arborescens and Caragana opulens
Source: BMC Genom Data. 2024 Feb 9;25:16. doi: 10.1186/s12863-024-01202-4 (PMC10854190; doi:10.1186/s12863-024-01202-4)
Supplement: Supplementary file 8 — Additional file 8: Table S4. Types and numbers of SSR in chloroplast genome of C. arborescens and C.opulens. [file 12863_2024_1202_MOESM8_ESM.doc]

Table S4 Types and numbers of SSR in chloroplast genome of *C. arborescens* and *C.opulens*

| Nucleotide | Type | Length (bp) | *C. arborescens* number | Ratio(%) | *C.opulens* number | Ratio (%) |
| --- | --- | --- | --- | --- | --- | --- |
| Mononucleotide | A/T | 8 | 65 | 57.04 | 68 | 63.40 |
|  |  | 9 | 41 |  | 46 |  |
|  |  | 10 | 29 |  | 29 |  |
|  |  | 11 | 11 |  | 13 |  |
|  |  | 12 | 9 |  | 5 |  |
|  |  | 13 | 1 |  | 4 |  |
|  |  | 14 | 1 |  | 1 |  |
|  |  | 15 | 1 |  | 1 |  |
|  | C | 11 | 0 |  | 1 |  |
| Dinucleotide | AT/AT | 5 | 13 | 7.58 | 10 | 4.91 |
|  |  | 6 | 7 |  | 1 |  |
|  |  | 7 | 0 |  | 1 |  |
|  | AG/CT | 5 | 1 |  | 1 |  |
| Trinucleotide | AAC/GTT | 3 | 4 | 29.24 | 5 | 28.68 |
|  | AAG/CTT | 3 | 19 |  | 25 |  |
|  |  | 4 | 1 |  | 1 |  |
|  | AAT/ATT | 3 | 34 |  | 22 |  |
|  |  | 4 | 4 |  | 3 |  |
|  |  | 5 | 1 |  | 1 |  |
|  | ACC/GGT | 3 | 2 |  | 2 |  |
|  | ACT/AGT | 3 | 4 |  | 4 |  |
|  | AGC/CTG | 3 | 5 |  | 5 |  |
|  | AGG/CCT | 3 | 0 |  | 1 |  |
|  | ATC/ATG | 3 | 7 |  | 7 |  |
| Tetranucleotide | AAAG/CTTT | 4 | 1 | 6.14 | 1 | 2.64 |
|  | AAAT/ATTT | 4 | 11 |  | 4 |  |
|  |  | 5 | 1 |  | 0 |  |
|  | AATT/AATT | 4 | 2 |  | 0 |  |
|  | ACAT/ATGT | 4 | 1 |  | 1 |  |
|  | ACCT/AGGT | 4 | 1 |  | 1 |  |
| Pentanucleotide | AATAT/ATATT | 5 | 0 | 0 | 1 | 0.38 |
