# Supplementary material for: Chloroplast genome analyses of Caragana arborescens and Caragana opulens
Source: BMC Genom Data. 2024 Feb 9;25:16. doi: 10.1186/s12863-024-01202-4 (PMC10854190; doi:10.1186/s12863-024-01202-4)
Supplement: Supplementary file 9 — Additional file 9: Table S5. Distribution of SSRs in cp genome of C. arborescens and C.opulens. [file 12863_2024_1202_MOESM9_ESM.doc]

Table S5 Distribution of SSRs in cp genome of *C. arborescens* and *C.opulens*.

| C. arborescens | | | | | C.opulens | | | | |
| --- | --- | --- | --- | --- | --- | --- | --- | --- | --- |
| Start Position on Genoem | End Position on Genoem | Repeat Motif | Length(bp) | Gene | Start Position on Genoem | End Position on Genoem | Repeat Motif | Length(bp) | Gene |
| 196 | 207 | (T)12 | 12 |  | 191 | 200 | (T)10 | 10 |  |
| 1724 | 1733 | (T)10 | 10 |  | 1716 | 1725 | (T)10 | 10 |  |
| 1755 | 1762 | (A)8 | 8 |  | 2230 | 2241 | (TCT)4 | 12 | matK |
| 2234 | 2242 | (CTT)3 | 9 | matK | 2369 | 2379 | (T)11 | 11 | matK |
| 2372 | 2382 | (T)11 | 11 | matK | 2913 | 2920 | (A)8 | 8 | matK |
| 2916 | 2923 | (A)8 | 8 | matK | 4028 | 4036 | (T)9 | 9 | trnK-UUU |
| 3887 | 3896 | (A)10 | 10 | trnK-UUU | 4435 | 4442 | (A)8 | 8 |  |
| 4034 | 4042 | (T)9 | 9 | trnK-UUU | 4526 | 4534 | (CTA)3 | 9 |  |
| 4671 | 4678 | (A)8 | 8 |  | 5093 | 5101 | (AGC)3 | 9 | rbcL |
| 4704 | 4725 | (ATT)3tttca(T)8 | 22 |  | 6504 | 6512 | (CAA)3 | 9 |  |
| 4905 | 4912 | (A)8 | 8 |  | 6697 | 6705 | (T)9 | 9 |  |
| 5128 | 5135 | (A)8 | 8 |  | 6837 | 6844 | (A)8 | 8 |  |
| 5475 | 5483 | (AGC)3 | 9 | rbcL | 6972 | 6979 | (A)8 | 8 |  |
| 6886 | 6894 | (CAA)3 | 9 |  | 7204 | 7213 | (A)10 | 10 |  |
| 7079 | 7088 | (T)10 | 10 |  | 7277 | 7284 | (A)8 | 8 | atpB |
| 7220 | 7227 | (A)8 | 8 |  | 9143 | 9152 | (A)10 | 10 |  |
| 7355 | 7362 | (A)8 | 8 |  | 9974 | 9982 | (CTT)3 | 9 | trnV-UAC |
| 7510 | 7518 | (TTA)3 | 9 |  | 10122 | 10130 | (A)9 | 9 | trnV-UAC |
| 7591 | 7602 | (A)12 | 12 |  | 10373 | 10381 | (T)9 | 9 |  |
| 7666 | 7673 | (A)8 | 8 | atpB | 10423 | 10431 | (TTA)3 | 9 |  |
| 9548 | 9556 | (A)9 | 9 |  | 12031 | 12040 | (TA)5 | 10 | ndhJ |
| 9869 | 9877 | (TAT)3 | 9 |  | 13227 | 13241 | (A)15 | 15 |  |
| 10376 | 10384 | (CTT)3 | 9 | trnV-UAC | 13257 | 13265 | (AGT)3 | 9 |  |
| 10524 | 10532 | (A)9 | 9 | trnV-UAC | 14494 | 14504 | (T)11 | 11 |  |
| 10808 | 10816 | (T)9 | 9 |  | 14694 | 14703 | (AT)5 | 10 |  |
| 10861 | 10882 | (TAT)4t(TTA)3 | 22 |  | 15626 | 15634 | (T)9 | 9 |  |
| 12453 | 12462 | (TA)5 | 10 | ndhJ | 15699 | 15706 | (T)8 | 8 |  |
| 12867 | 12874 | (T)8 | 8 |  | 16885 | 16893 | (A)9 | 9 |  |
| 13660 | 13670 | (A)11 | 11 |  | 17195 | 17203 | (T)9 | 9 | ycf3 |
| 13686 | 13694 | (AGT)3 | 9 |  | 17249 | 17256 | (A)8 | 8 | ycf3 |
| 14926 | 14934 | (T)9 | 9 |  | 17495 | 17502 | (A)8 | 8 | ycf3 |
| 15132 | 15143 | (AT)6 | 12 |  | 18182 | 18189 | (A)8 | 8 | ycf3 |
| 15362 | 15373 | (AT)6 | 12 |  | 18297 | 18304 | (T)8 | 8 | ycf3 |
| 15475 | 15484 | (AT)5 | 10 |  | 18622 | 18630 | (TTC)3 | 9 | ycf3 |
| 15515 | 15523 | (ATA)3 | 9 |  | 18662 | 18670 | (CAT)3 | 9 | ycf3 |
| 15549 | 15568 | (ATA)3agc(A)8 | 20 |  | 18800 | 18807 | (A)8 | 8 | ycf3 |
| 16323 | 16331 | (T)9 | 9 |  | 18884 | 18891 | (A)8 | 8 | ycf3 |
| 17595 | 17603 | (A)9 | 9 |  | 19275 | 19282 | (T)8 | 8 |  |
| 17896 | 17904 | (T)9 | 9 | ycf3 | 20139 | 20147 | (TAC)3 | 9 | psaA |
| 18194 | 18201 | (A)8 | 8 | ycf3 | 20893 | 20901 | (CAT)3 | 9 | psaA |
| 18894 | 18903 | (A)10 | 10 | ycf3 | 21219 | 21227 | (TGC)3 | 9 | psaA |
| 19011 | 19018 | (T)8 | 8 | ycf3 | 23117 | 23125 | (CAT)3 | 9 | psaB |
| 19376 | 19384 | (CAT)3 | 9 | ycf3 | 23413 | 23421 | (TCA)3 | 9 | psaB |
| 19515 | 19522 | (A)8 | 8 | ycf3 | 24646 | 24654 | (AGA)3 | 9 | rps14 |
| 19985 | 19993 | (T)9 | 9 |  | 25180 | 25188 | (AAG)3 | 9 |  |
| 20210 | 20220 | (T)11 | 11 |  | 25848 | 25856 | (ATA)3 | 9 |  |
| 20872 | 20880 | (TAC)3 | 9 | psaA | 26339 | 26347 | (T)9 | 9 |  |
| 21626 | 21634 | (CAT)3 | 9 | psaA | 26381 | 26392 | (AT)6 | 12 |  |
| 21952 | 21960 | (TGC)3 | 9 | psaA | 26727 | 26735 | (A)9 | 9 |  |
| 23850 | 23858 | (CAT)3 | 9 | psaB | 29658 | 29665 | (A)8 | 8 |  |
| 24146 | 24154 | (TCA)3 | 9 | psaB | 29870 | 29878 | (T)9 | 9 |  |
| 25385 | 25393 | (AGA)3 | 9 | rps14 | 30385 | 30393 | (T)9 | 9 |  |
| 25919 | 25930 | (AAG)4 | 12 |  | 30472 | 30481 | (AT)5 | 10 |  |
| 26308 | 26337 | (TAT)3atttattta(AT)6 | 30 |  | 30630 | 30640 | (T)11 | 11 |  |
| 26414 | 26425 | (AATA)3 | 12 |  | 30720 | 30728 | (A)9 | 9 |  |
| 26447 | 26455 | (ATA)3 | 9 |  | 30883 | 30892 | (TA)5 | 10 |  |
| 26944 | 26953 | (T)10 | 10 |  | 31083 | 31091 | (A)9 | 9 |  |
| 26987 | 26996 | (AT)5 | 10 |  | 31323 | 31331 | (T)9 | 9 |  |
| 30273 | 30280 | (A)8 | 8 |  | 31915 | 31939 | (A)12gagtc(T)8 | 25 |  |
| 30420 | 30427 | (A)8 | 8 |  | 32120 | 32131 | (TATG)3 | 12 |  |
| 30491 | 30498 | (T)8 | 8 |  | 32323 | 32334 | (TTTC)3 | 12 |  |
| 30543 | 30551 | (TAA)3 | 9 |  | 32382 | 32389 | (T)8 | 8 |  |
| 31013 | 31021 | (T)9 | 9 |  | 33583 | 33591 | (TAA)3 | 9 |  |
| 31100 | 31109 | (AT)5 | 10 |  | 33873 | 33880 | (T)8 | 8 |  |
| 31254 | 31264 | (T)11 | 11 |  | 33904 | 33912 | (ATA)3 | 9 |  |
| 31523 | 31532 | (TA)5 | 10 |  | 34389 | 34398 | (AT)5 | 10 |  |
| 31767 | 31776 | (T)10 | 10 |  | 34875 | 34886 | (A)12 | 12 |  |
| 32379 | 32403 | (A)12gagtc(T)8 | 25 |  | 35057 | 35066 | (A)10 | 10 |  |
| 32497 | 32506 | (AT)5 | 10 |  | 35507 | 35514 | (A)8 | 8 |  |
| 32598 | 32609 | (TATG)3 | 12 |  | 36873 | 36880 | (A)8 | 8 | rpoB |
| 32719 | 32726 | (A)8 | 8 |  | 38544 | 38553 | (AT)5 | 10 | rpoB |
| 32800 | 32811 | (TTTC)3 | 12 |  | 39818 | 39826 | (ATT)3 | 9 | rpoC1 |
| 33763 | 33774 | (ATTA)3 | 12 |  | 40022 | 40030 | (A)9 | 9 | rpoC1 |
| 34059 | 34067 | (TAA)3 | 9 |  | 40063 | 40072 | (T)10 | 10 | rpoC1 |
| 34349 | 34356 | (T)8 | 8 |  | 40363 | 40371 | (A)9 | 9 | rpoC1 |
| 34380 | 34388 | (ATA)3 | 9 |  | 40488 | 40495 | (T)8 | 8 | rpoC1 |
| 34524 | 34531 | (A)8 | 8 |  | 40878 | 40886 | (GAA)3 | 9 | rpoC1 |
| 34774 | 34781 | (A)8 | 8 |  | 41894 | 41915 | (A)9gaacc(T)8 | 22 | rpoC1 |
| 34872 | 34880 | (TAA)3 | 9 |  | 43161 | 43170 | (AT)5 | 10 | rpoC2 |
| 35151 | 35159 | (AGT)3 | 9 |  | 43924 | 43932 | (A)9 | 9 | rpoC2 |
| 35336 | 35345 | (A)10 | 10 |  | 44399 | 44407 | (T)9 | 9 | rpoC2 |
| 36121 | 36128 | (A)8 | 8 |  | 44540 | 44552 | (A)13 | 13 | rpoC2 |
| 37325 | 37333 | (A)9 | 9 | rpoB | 44647 | 44656 | (A)10 | 10 | rpoC2 |
| 37430 | 37437 | (A)8 | 8 | rpoB | 44668 | 44675 | (A)8 | 8 | rpoC2 |
| 39101 | 39110 | (AT)5 | 10 | rpoB | 45723 | 45731 | (TAA)3 | 9 | rpoC2 |
| 39998 | 40005 | (A)8 | 8 | rpoC1 | 46464 | 46473 | (A)10 | 10 | rpoC2 |
| 40579 | 40597 | (A)8gac(T)8 | 19 | rpoC1 | 46790 | 46815 | (T)9caatc(T)12 | 26 |  |
| 40620 | 40628 | (T)9 | 9 | rpoC1 | 47061 | 47069 | (A)9 | 9 | rps2 |
| 40918 | 40927 | (A)10 | 10 | rpoC1 | 47452 | 47460 | (ATG)3 | 9 | rps2 |
| 41043 | 41051 | (T)9 | 9 | rpoC1 | 47545 | 47553 | (TAA)3 | 9 |  |
| 41434 | 41442 | (GAA)3 | 9 | rpoC1 | 47572 | 47579 | (T)8 | 8 |  |
| 42450 | 42471 | (A)9gaacc(T)8 | 22 | rpoC1 | 47639 | 47648 | (A)10 | 10 |  |
| 43717 | 43726 | (AT)5 | 10 | rpoC2 | 47756 | 47763 | (T)8 | 8 |  |
| 44955 | 44963 | (T)9 | 9 | rpoC2 | 48384 | 48392 | (GTT)3 | 9 | atpI |
| 45096 | 45108 | (A)13 | 13 | rpoC2 | 48546 | 48554 | (T)9 | 9 |  |
| 45203 | 45212 | (A)10 | 10 | rpoC2 | 48671 | 48678 | (C)8 | 8 |  |
| 45224 | 45231 | (A)8 | 8 | rpoC2 | 48735 | 48743 | (ATA)3 | 9 |  |
| 46273 | 46281 | (TAA)3 | 9 | rpoC2 | 49185 | 49195 | (A)11 | 11 |  |
| 47014 | 47023 | (A)10 | 10 | rpoC2 | 49652 | 49662 | (T)11 | 11 |  |
| 47331 | 47364 | (T)10caatc(T)9(A)10 | 34 |  | 49965 | 49973 | (TCT)3 | 9 |  |
| 47605 | 47613 | (A)9 | 9 | rps2 | 50119 | 50127 | (A)9 | 9 |  |
| 47996 | 48004 | (ATG)3 | 9 | rps2 | 50322 | 50329 | (T)8 | 8 | atpF |
| 48089 | 48097 | (TAA)3 | 9 |  | 50777 | 50786 | (A)10 | 10 | atpF |
| 48183 | 48192 | (A)10 | 10 |  | 51492 | 51500 | (T)9 | 9 |  |
| 48927 | 48935 | (GTT)3 | 9 | atpI | 53168 | 53176 | (A)9 | 9 |  |
| 49089 | 49097 | (T)9 | 9 |  | 53770 | 53778 | (A)9 | 9 | trnG-UCC |
| 49156 | 49164 | (A)9 | 9 |  | 54729 | 54736 | (A)8 | 8 |  |
| 49278 | 49286 | (ATA)3 | 9 |  | 54754 | 54764 | (A)11 | 11 |  |
| 49314 | 49323 | (A)10 | 10 |  | 55034 | 55041 | (T)8 | 8 |  |
| 49859 | 49868 | (T)10 | 10 |  | 55295 | 55305 | (A)11 | 11 |  |
| 50209 | 50218 | (T)10 | 10 |  | 55495 | 55503 | (TGG)3 | 9 |  |
| 50361 | 50369 | (T)9 | 9 |  | 55964 | 55988 | (T)10attgta(TAT)3 | 25 |  |
| 50739 | 50746 | (A)8 | 8 |  | 56296 | 56305 | (A)10 | 10 |  |
| 50941 | 50948 | (T)8 | 8 | atpF | 56813 | 56823 | (A)11 | 11 |  |
| 51388 | 51396 | (A)9 | 9 | atpF | 56886 | 56906 | (A)8gaa(T)10 | 21 |  |
| 53752 | 53759 | (A)8 | 8 |  | 56924 | 56932 | (TTA)3 | 9 |  |
| 54046 | 54057 | (TAAA)3 | 12 |  | 57517 | 57525 | (TGA)3 | 9 | accD |
| 54407 | 54415 | (A)9 | 9 | trnG-UCC | 58630 | 58639 | (T)10 | 10 |  |
| 55007 | 55018 | (ATT)4 | 12 |  | 58695 | 58703 | (ATA)3 | 9 |  |
| 55339 | 55347 | (ATT)3 | 9 |  | 59060 | 59068 | (ATA)3 | 9 |  |
| 55397 | 55406 | (A)10 | 10 |  | 59359 | 59366 | (A)8 | 8 |  |
| 55663 | 55670 | (T)8 | 8 |  | 60158 | 60166 | (A)9 | 9 | ycf4 |
| 55924 | 55934 | (A)11 | 11 |  | 60262 | 60270 | (TTC)3 | 9 |  |
| 56127 | 56135 | (TGG)3 | 9 |  | 61859 | 61867 | (A)9 | 9 | petA |
| 56593 | 56616 | (T)9attgta(TAT)3 | 24 |  | 62033 | 62041 | (TAA)3 | 9 | petA |
| 56669 | 56677 | (ATT)3 | 9 |  | 62613 | 62625 | (T)13 | 13 |  |
| 57488 | 57498 | (A)11 | 11 |  | 64268 | 64276 | (TAT)3 | 9 |  |
| 57561 | 57568 | (T)8 | 8 |  | 64726 | 64734 | (T)9 | 9 |  |
| 57586 | 57594 | (TTA)3 | 9 |  | 64928 | 64936 | (A)9 | 9 |  |
| 58174 | 58182 | (TGA)3 | 9 | accD | 65124 | 65131 | (A)8 | 8 |  |
| 59287 | 59298 | (T)12 | 12 |  | 66533 | 66542 | (T)10 | 10 |  |
| 59354 | 59362 | (ATA)3 | 9 |  | 66582 | 66589 | (A)8 | 8 |  |
| 59510 | 59517 | (T)8 | 8 |  | 66637 | 66645 | (ATA)3 | 9 |  |
| 59573 | 59584 | (AT)6 | 12 |  | 66700 | 66707 | (A)8 | 8 |  |
| 59714 | 59722 | (ATA)3 | 9 |  | 66817 | 66836 | (TATAT)3(TA)5* | 20 |  |
| 60022 | 60031 | (A)10 | 10 |  | 67007 | 67014 | (A)8 | 8 |  |
| 60823 | 60830 | (A)8 | 8 | ycf4 | 67230 | 67256 | (CTT)3tcatttc(T)11 | 27 |  |
| 60916 | 60924 | (TTC)3 | 9 |  | 67285 | 67296 | (A)12 | 12 |  |
| 61079 | 61088 | (AT)5 | 10 |  | 67611 | 67619 | (TTC)3 | 9 |  |
| 62502 | 62510 | (A)9 | 9 | petA | 67968 | 67979 | (TAT)4 | 12 |  |
| 62676 | 62684 | (TAA)3 | 9 | petA | 68095 | 68102 | (A)8 | 8 |  |
| 62715 | 62722 | (A)8 | 8 | petA | 68342 | 68350 | (AAC)3 | 9 | rps18 |
| 63249 | 63262 | (T)14 | 14 |  | 68448 | 68457 | (A)10 | 10 | rps18 |
| 63907 | 63914 | (A)8 | 8 |  | 68485 | 68512 | (ACT)3gtttt(A)14 | 28 | rps18 |
| 65339 | 65350 | (T)12 | 12 |  | 68653 | 68660 | (T)8 | 8 |  |
| 65544 | 65551 | (A)8 | 8 |  | 69292 | 69299 | (T)8 | 8 |  |
| 65739 | 65746 | (A)8 | 8 |  | 69898 | 69905 | (T)8 | 8 |  |
| 65860 | 65871 | (TATT)3 | 12 |  | 70880 | 70888 | (ATT)3 | 9 |  |
| 66490 | 66497 | (T)8 | 8 |  | 70970 | 70978 | (T)9 | 9 |  |
| 67172 | 67181 | (T)10 | 10 |  | 71280 | 71291 | (ATAA)3 | 12 |  |
| 67273 | 67304 | (ATA)4tatagagt(ATA)4 | 32 |  | 71779 | 71788 | (T)10 | 10 | clpP |
| 67367 | 67374 | (A)8 | 8 |  | 72185 | 72192 | (T)8 | 8 | clpP |
| 67527 | 67538 | (TA)6 | 12 |  | 72273 | 72280 | (T)8 | 8 | clpP |
| 67933 | 67960 | (CTT)3tcatttc(T)12 | 28 |  | 72359 | 72366 | (T)8 | 8 | clpP |
| 67979 | 67987 | (A)9 | 9 |  | 72442 | 72449 | (T)8 | 8 | clpP |
| 68797 | 68804 | (A)8 | 8 |  | 72525 | 72532 | (T)8 | 8 | clpP |
| 69132 | 69141 | (A)10 | 10 | rps18 | 72695 | 72703 | (A)9 | 9 | clpP |
| 69169 | 69198 | (ACT)3gttttt(A)15 | 30 | rps18 | 72772 | 72781 | (T)10 | 10 | clpP |
| 69337 | 69347 | (T)11 | 11 |  | 73118 | 73126 | (CAT)3 | 9 | clpP |
| 69972 | 69979 | (T)8 | 8 |  | 73288 | 73296 | (A)9 | 9 |  |
| 70595 | 70603 | (T)9 | 9 |  | 73383 | 73391 | (CCA)3 | 9 |  |
| 72001 | 72012 | (T)12 | 12 | clpP | 74348 | 74356 | (TTG)3 | 9 | psbB |
| 72043 | 72050 | (T)8 | 8 | clpP | 75163 | 75171 | (T)9 | 9 |  |
| 72211 | 72234 | (A)11tta(T)10 | 24 | clpP | 76296 | 76303 | (A)8 | 8 | petB |
| 72295 | 72303 | (T)9 | 9 | clpP | 76413 | 76420 | (T)8 | 8 | petB |
| 72640 | 72648 | (CAT)3 | 9 | clpP | 76582 | 76593 | (AAT)4 | 12 | petB |
| 72935 | 72943 | (CCA)3 | 9 |  | 77660 | 77672 | (T)13 | 13 |  |
| 73900 | 73908 | (TTG)3 | 9 | psbB | 77902 | 77910 | (GGA)3 | 9 | petD |
| 74715 | 74723 | (T)9 | 9 |  | 79489 | 79497 | (TTC)3 | 9 | rpoA |
| 75849 | 75856 | (A)8 | 8 | petB | 81238 | 81245 | (A)8 | 8 |  |
| 77204 | 77213 | (T)10 | 10 |  | 81448 | 81455 | (T)8 | 8 |  |
| 78704 | 78711 | (A)8 | 8 |  | 82097 | 82105 | (ATA)3 | 9 |  |
| 79026 | 79034 | (TTC)3 | 9 | rpoA | 82171 | 82180 | (A)10 | 10 |  |
| 81649 | 81657 | (ATA)3 | 9 |  | 82260 | 82267 | (T)8 | 8 |  |
| 81723 | 81732 | (A)10 | 10 |  | 82310 | 82319 | (AT)5 | 10 |  |
| 81813 | 81820 | (A)8 | 8 |  | 82578 | 82586 | (TTA)3 | 9 | rpl14 |
| 81851 | 81860 | (AT)5 | 10 |  | 82689 | 82697 | (CAA)3 | 9 | rpl14 |
| 81887 | 81895 | (TAT)3 | 9 |  | 82814 | 82823 | (T)10 | 10 |  |
| 82358 | 82366 | (TAT)3 | 9 | rpl14 | 82868 | 82876 | (A)9 | 9 |  |
| 82468 | 82476 | (CAA)3 | 9 | rpl14 | 84420 | 84427 | (A)8 | 8 | rpl16 |
| 82593 | 82601 | (T)9 | 9 |  | 84552 | 84559 | (T)8 | 8 |  |
| 82626 | 82633 | (A)8 | 8 |  | 84948 | 84955 | (T)8 | 8 | rps3 |
| 82646 | 82657 | (A)12 | 12 |  | 85502 | 85510 | (TGC)3 | 9 |  |
| 84142 | 84149 | (A)8 | 8 | rpl16 | 85526 | 85534 | (A)9 | 9 |  |
| 84274 | 84283 | (T)10 | 10 |  | 85793 | 85801 | (T)9 | 9 | rps19 |
| 84672 | 84679 | (T)8 | 8 | rps3 | 85869 | 85877 | (A)9 | 9 |  |
| 85176 | 85184 | (TGC)3 | 9 |  | 86179 | 86188 | (T)10 | 10 |  |
| 85200 | 85208 | (A)9 | 9 |  | 86262 | 86271 | (T)10 | 10 |  |
| 85393 | 85401 | (TTA)3 | 9 | rps19 | 86493 | 86501 | (AAG)3 | 9 |  |
| 85467 | 85475 | (T)9 | 9 | rps19 | 86668 | 86677 | (T)10 | 10 |  |
| 86092 | 86100 | (AGA)3 | 9 |  | 86904 | 86913 | (T)10 | 10 |  |
| 86328 | 86336 | (CTT)3 | 9 | rpl2 | 86987 | 86999 | (T)13 | 13 |  |
| 88138 | 88146 | (T)9 | 9 |  | 87115 | 87123 | (CTT)3 | 9 | rpl2 |
| 88323 | 88331 | (TAA)3 | 9 |  | 89009 | 89017 | (T)9 | 9 |  |
| 88351 | 88362 | (ATTT)3 | 12 |  | 89222 | 89233 | (AATA)3 | 12 |  |
| 88376 | 88387 | (ATTT)3 | 12 |  | 89958 | 89967 | (A)10 | 10 |  |
| 88401 | 88412 | (ATTT)3 | 12 |  | 90658 | 90666 | (AAG)3 | 9 | ycf2 |
| 88426 | 88437 | (ATTT)3 | 12 |  | 92274 | 92282 | (A)9 | 9 | ycf2 |
| 88451 | 88462 | (ATTT)3 | 12 |  | 93090 | 93098 | (CTT)3 | 9 | ycf2 |
| 88532 | 88543 | (ATTT)3 | 12 |  | 93392 | 93400 | (GAA)3 | 9 | ycf2 |
| 88621 | 88629 | (TAA)3 | 9 |  | 93765 | 93772 | (A)8 | 8 | ycf2 |
| 89663 | 89671 | (AAG)3 | 9 | ycf2 | 94877 | 94885 | (GAA)3 | 9 | ycf2 |
| 91288 | 91296 | (A)9 | 9 | ycf2 | 96605 | 96612 | (A)8 | 8 |  |
| 92104 | 92112 | (CTT)3 | 9 | ycf2 | 97624 | 97632 | (AGA)3 | 9 | ndhB |
| 92400 | 92408 | (GAA)3 | 9 | ycf2 | 99068 | 99076 | (AGA)3 | 9 | ndhB |
| 93885 | 93893 | (GAA)3 | 9 | ycf2 | 99968 | 99976 | (T)9 | 9 | rps7 |
| 96585 | 96593 | (AGA)3 | 9 | ndhB | 100522 | 100530 | (T)9 | 9 |  |
| 98029 | 98037 | (AGA)3 | 9 | ndhB | 106928 | 106936 | (CTG)3 | 9 | rrn23 |
| 98926 | 98934 | (T)9 | 9 | rps7 | 108442 | 108453 | (AGGT)3 | 12 | rrn23 |
| 105171 | 105179 | (CTG)3 | 9 | rrn23 | 110384 | 110393 | (AG)5 | 10 |  |
| 106685 | 106696 | (AGGT)3 | 12 | rrn23 | 111968 | 111979 | (ATA)4 | 12 |  |
| 108533 | 108541 | (A)9 | 9 |  | 113099 | 113107 | (T)9 | 9 | ycf1 |
| 108640 | 108649 | (AG)5 | 10 |  | 113187 | 113195 | (A)9 | 9 | ycf1 |
| 108949 | 108956 | (A)8 | 8 |  | 113602 | 113611 | (T)10 | 10 | ycf1 |
| 110025 | 110039 | (ATA)5 | 15 |  | 113712 | 113720 | (A)9 | 9 | ycf1 |
| 111166 | 111174 | (T)9 | 9 | ycf1 | 114324 | 114331 | (A)8 | 8 | ycf1 |
| 111254 | 111262 | (A)9 | 9 | ycf1 | 114411 | 114419 | (TAA)3 | 9 | ycf1 |
| 111669 | 111678 | (T)10 | 10 | ycf1 | 114684 | 114695 | (A)12 | 12 | ycf1 |
| 111789 | 111798 | (A)10 | 10 | ycf1 | 115390 | 115397 | (A)8 | 8 | ycf1 |
| 112391 | 112398 | (A)8 | 8 | ycf1 | 117416 | 117425 | (AT)5 | 10 |  |
| 112478 | 112486 | (TAA)3 | 9 | ycf1 | 117524 | 117532 | (T)9 | 9 |  |
| 112751 | 112796 | (A)12gatctactg(A)10cgtg(A)11 | 46 | ycf1 | 117608 | 117622 | (TAA)5 | 15 |  |
| 113059 | 113066 | (A)8 | 8 | ycf1 | 117633 | 117643 | (A)11 | 11 |  |
| 113460 | 113467 | (A)8 | 8 | ycf1 | 117866 | 117875 | (A)10 | 10 |  |
| 115432 | 115441 | (AT)5 | 10 |  | 118040 | 118048 | (TTC)3 | 9 |  |
| 115470 | 115479 | (TA)5 | 10 |  | 118264 | 118277 | (AT)7 | 14 |  |
| 115576 | 115583 | (T)8 | 8 |  | 119140 | 119148 | (TTC)3 | 9 |  |
| 115659 | 115684 | (TAA)3caaatttg(A)9 | 26 |  | 119372 | 119381 | (A)10 | 10 |  |
| 115874 | 115891 | (A)11(AAG)3* | 18 | rps15 | 119460 | 119477 | (A)11(AAG)3* | 18 | rps15 |
| 116170 | 116177 | (T)8 | 8 |  | 119756 | 119763 | (T)8 | 8 |  |
| 117885 | 117893 | (GCT)3 | 9 | ndhA | 121471 | 121479 | (GCT)3 | 9 | ndhA |
| 119274 | 119282 | (GAA)3 | 9 | ndhA | 122859 | 122867 | (GAA)3 | 9 | ndhA |
| 120226 | 120241 | (AAAT)4 | 16 | ndhI | 123811 | 123822 | (AAAT)3 | 12 | ndhI |
| 120283 | 120292 | (AT)5 | 10 |  | 124026 | 124033 | (T)8 | 8 |  |
| 120337 | 120345 | (A)9 | 9 |  | 124115 | 124122 | (T)8 | 8 |  |
| 120359 | 120367 | (A)9 | 9 |  | 124762 | 124769 | (T)8 | 8 |  |
| 120503 | 120514 | (TA)6 | 12 |  | 126749 | 126756 | (A)8 | 8 | ndhD |
| 120620 | 120627 | (T)8 | 8 |  | 127045 | 127052 | (T)8 | 8 | ndhD |
| 121356 | 121363 | (T)8 | 8 |  | 127136 | 127147 | (TATT)3 | 12 | ndhD |
| 123343 | 123350 | (A)8 | 8 | ndhD | 127940 | 127948 | (AGA)3 | 9 | ccsA |
| 123730 | 123741 | (TATT)3 | 12 | ndhD | 128651 | 128659 | (CTT)3 | 9 |  |
| 124139 | 124150 | (TA)6 | 12 |  | 129513 | 129520 | (A)8 | 8 | rpl32 |
| 124537 | 124545 | (AGA)3 | 9 | ccsA | 129587 | 129594 | (T)8 | 8 | rpl32 |
| 125434 | 125445 | (TATT)3 | 12 |  | 129612 | 129622 | (T)11 | 11 | rpl32 |
| 125630 | 125638 | (TTA)3 | 9 |  | 129768 | 129775 | (T)8 | 8 |  |
| 125932 | 125943 | (TTAA)3 | 12 |  | 129916 | 129923 | (A)8 | 8 |  |
| 126154 | 126161 | (A)8 | 8 | rpl32 | 130062 | 130070 | (TTA)3 | 9 |  |
| 126228 | 126235 | (T)8 | 8 | rpl32 | 130343 | 130353 | (T)11 | 11 |  |
| 126253 | 126263 | (T)11 | 11 | rpl32 | 130429 | 130437 | (CTT)3 | 9 | ndhF |
| 126409 | 126418 | (T)10 | 10 |  | 130918 | 130926 | (TTA)3 | 9 | ndhF |
| 126570 | 126577 | (A)8 | 8 |  | 131021 | 131028 | (T)8 | 8 | ndhF |
| 126716 | 126724 | (TTA)3 | 9 |  | 131802 | 131809 | (A)8 | 8 | ndhF |
| 127004 | 127012 | (T)9 | 9 |  | 131851 | 131859 | (AAT)3 | 9 | ndhF |
| 127090 | 127098 | (CTT)3 | 9 | ndhF | 131973 | 131980 | (T)8 | 8 | ndhF |
| 127579 | 127587 | (TTA)3 | 9 | ndhF | 132541 | 132548 | (T)8 | 8 | ndhF |
| 128463 | 128472 | (A)10 | 10 | ndhF | 132781 | 132790 | (T)10 | 10 |  |
| 128512 | 128520 | (AAT)3 | 9 | ndhF |  |  |  |  |  |
| 128634 | 128641 | (T)8 | 8 | ndhF |  |  |  |  |  |
| 129202 | 129209 | (T)8 | 8 | ndhF |  |  |  |  |  |
