# Supplementary material for: Chloroplast genome analyses of Caragana arborescens and Caragana opulens
Source: BMC Genom Data. 2024 Feb 9;25:16. doi: 10.1186/s12863-024-01202-4 (PMC10854190; doi:10.1186/s12863-024-01202-4)
Supplement: Supplementary file 10 — Additional file 10: Fig. S5. The number of SSRs was found in coding (CDS), and intronic regions, intergenic (IGS), Respectively. [file 12863_2024_1202_MOESM10_ESM.doc]

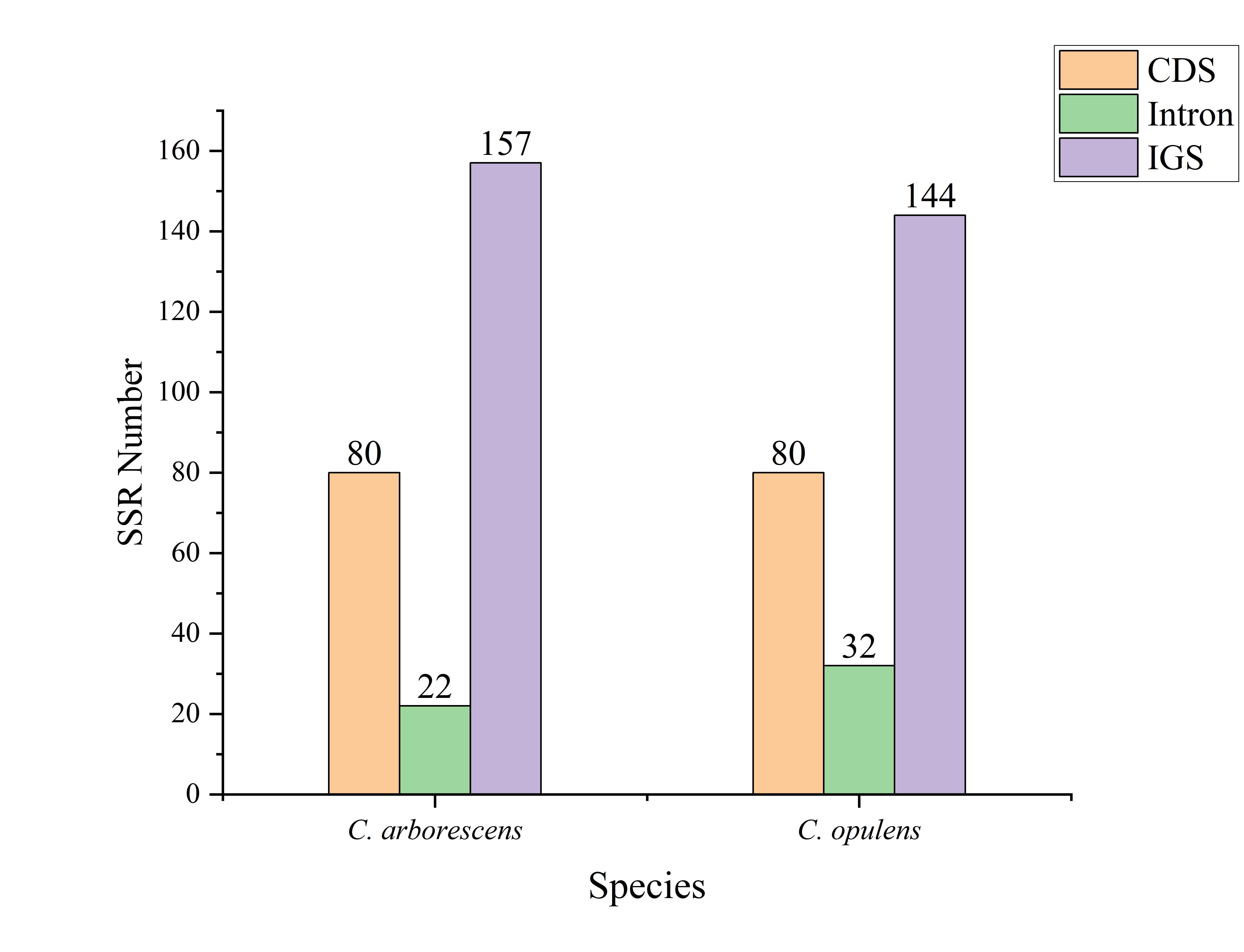


**Fig. S5** The number of SSRs was found in coding (CDS), and intronic regions, intergenic (IGS), Respectively
