# Supplementary material for: Chloroplast genome analyses of Caragana arborescens and Caragana opulens
Source: BMC Genom Data. 2024 Feb 9;25:16. doi: 10.1186/s12863-024-01202-4 (PMC10854190; doi:10.1186/s12863-024-01202-4)
Supplement: Supplementary file 11 — Additional file 11: Table S6. Analysis of coding ability and codon preference of chloroplast genome. [file 12863_2024_1202_MOESM11_ESM.doc]

Table S6 Analysis of coding ability and codon preference of chloroplast genome

| Amino acid | Codon | *C.arborescens* | | | | *C.opulens* | | | |
| --- | --- | --- | --- | --- | --- | --- | --- | --- | --- |
|  |  | Number | RSCU | Total No. | Ratio (%) | Number | RSCU | Total No. | Ratio (%) |
| A（Ala） | GCA | 334 | 1.1 | 1 212 | 5.51 | 336 | 1.1036 | 1218 | 5.53 |
|  | GCC | 184 | 0.61 |  |  | 183 | 0.6008 |  |  |
|  | GCG | 124 | 0.41 |  |  | 126 | 0.4136 |  |  |
|  | GCU | 570 | 1.88 |  |  | 573 | 1.8816 |  |  |
| C（Cys） | UGC | 59 | 0.46 | 257 | 1.17 | 59 | 0.4522 | 261 | 1.18 |
|  | UGU | 198 | 1.54 |  |  | 202 | 1.5478 |  |  |
| D（Asp） | GAC | 166 | 0.38 | 877 | 3.99 | 166 | 0.3768 | 881 | 4.00 |
|  | GAU | 711 | 1.62 |  |  | 715 | 1.6232 |  |  |
| E（Glu） | GAA | 889 | 1.54 | 1 158 | 5.26 | 886 | 1.5172 | 1 168 | 5.30 |
|  | GAG | 269 | 0.46 |  |  | 282 | 0.4828 |  |  |
| F（Phe） | UUC | 399 | 0.62 | 1 291 | 5.87 | 398 | 0.6152 | 1 294 | 5.87 |
|  | UUU | 892 | 1.38 |  |  | 896 | 1.3848 |  |  |
| G（Gly） | GGA | 624 | 1.65 | 1 509 | 6.86 | 629 | 1.6628 | 1513 | 6.87 |
|  | GGC | 129 | 0.34 |  |  | 128 | 0.3384 |  |  |
|  | GGG | 218 | 0.58 |  |  | 217 | 0.5736 |  |  |
|  | GGU | 538 | 1.43 |  |  | 539 | 1.4248 |  |  |
| H（His） | CAC | 112 | 0.44 | 508 | 2.31 | 111 | 0.4328 | 513 | 2.33 |
|  | CAU | 396 | 1.56 |  |  | 402 | 1.5672 |  |  |
| I（Ile） | AUA | 631 | 0.96 | 1 979 | 9.00 | 626 | 0.9588 | 1 959 | 8.89 |
|  | AUC | 378 | 0.57 |  |  | 390 | 0.5973 |  |  |
|  | AUU | 970 | 1.47 |  |  | 943 | 1.4442 |  |  |
| K（Lys） | AAA | 920 | 1.55 | 1 186 | 5.39 | 928 | 1.548 | 1 199 | 5.44 |
|  | AAG | 266 | 0.45 |  |  | 271 | 0.452 |  |  |
| L（Leu） | CUA | 307 | 0.79 | 2 327 | 10.58 | 308 | 0.7872 | 2 347 | 10.65 |
|  | CUC | 139 | 0.36 |  |  | 143 | 0.3654 |  |  |
|  | CUG | 137 | 0.35 |  |  | 137 | 0.3504 |  |  |
|  | CUU | 472 | 1.22 |  |  | 488 | 1.2474 |  |  |
|  | UUA | 787 | 2.03 |  |  | 786 | 2.0094 |  |  |
|  | UUG | 485 | 1.25 |  |  | 485 | 1.2396 |  |  |
| M（Met） | AUC | 1 | 0.01 | 501 | 2.28 | 1 | 0.006 | 506 | 2.30 |
|  | AUG | 499 | 2.99 |  |  | 503 | 2.9823 |  |  |
|  | AUU | 1 | 0.01 |  |  | 2 | 0.012 |  |  |
| N（Asn） | AAC | 233 | 0.44 | 1 064 | 4.84 | 237 | 0.4506 | 1 052 | 4.77 |
|  | AAU | 831 | 1.56 |  |  | 815 | 1.5494 |  |  |
| P（Pro） | CCA | 270 | 1.2 | 902 | 4.10 | 272 | 1.2088 | 900 | 4.08 |
|  | CCC | 175 | 0.78 |  |  | 171 | 0.76 |  |  |
|  | CCG | 102 | 0.45 |  |  | 101 | 0.4488 |  |  |
|  | CCU | 355 | 1.57 |  |  | 356 | 1.5824 |  |  |
| Q（Gln） | CAA | 630 | 1.6 | 787 | 3.58 | 631 | 1.6056 | 786 | 3.57 |
|  | CAG | 157 | 0.4 |  |  | 155 | 0.3944 |  |  |
| R（Arg） | AGA | 376 | 1.79 | 1 259 | 5.72 | 373 | 1.7802 | 1 257 | 5.70 |
|  | AGG | 129 | 0.62 |  |  | 136 | 0.6492 |  |  |
|  | CGA | 292 | 1.39 |  |  | 294 | 1.4034 |  |  |
|  | CGC | 86 | 0.41 |  |  | 85 | 0.4056 |  |  |
|  | CGG | 91 | 0.43 |  |  | 88 | 0.42 |  |  |
|  | CGU | 285 | 1.36 |  |  | 281 | 1.341 |  |  |
| S（Ser） | AGC | 97 | 0.36 | 1 636 | 7.44 | 99 | 0.3642 | 1 631 | 7.40 |
|  | AGU | 351 | 1.29 |  |  | 350 | 1.2876 |  |  |
|  | UCA | 327 | 1.2 |  |  | 323 | 1.188 |  |  |
|  | UCC | 233 | 0.85 |  |  | 236 | 0.8682 |  |  |
|  | UCG | 156 | 0.57 |  |  | 152 | 0.5592 |  |  |
|  | UCU | 472 | 1.73 |  |  | 471 | 1.7328 |  |  |
| T（Thr） | ACA | 352 | 1.25 | 1 130 | 5.14 | 352 | 1.2384 | 1 137 | 5.16 |
|  | ACC | 191 | 0.68 |  |  | 199 | 0.7 |  |  |
|  | ACG | 116 | 0.41 |  |  | 117 | 0.4116 |  |  |
|  | ACU | 471 | 1.67 |  |  | 469 | 1.65 |  |  |
| V（Val） | GUA | 461 | 1.53 | 1 208 | 5.49 | 458 | 1.504 | 1 218 | 5.53 |
|  | GUC | 134 | 0.44 |  |  | 146 | 0.4796 |  |  |
|  | GUG | 153 | 0.51 |  |  | 155 | 0.5092 |  |  |
|  | GUU | 460 | 1.52 |  |  | 459 | 1.5072 |  |  |
| W（Trp） | UGG | 375 | 1 | 375 | 1.70 | 374 | 1 | 374 | 1.70 |
| Y（Tyr） | UAC | 149 | 0.36 | 832 | 3.78 | 149 | 0.363 | 821 | 3.73 |
|  | UAU | 683 | 1.64 |  |  | 672 | 1.637 |  |  |
